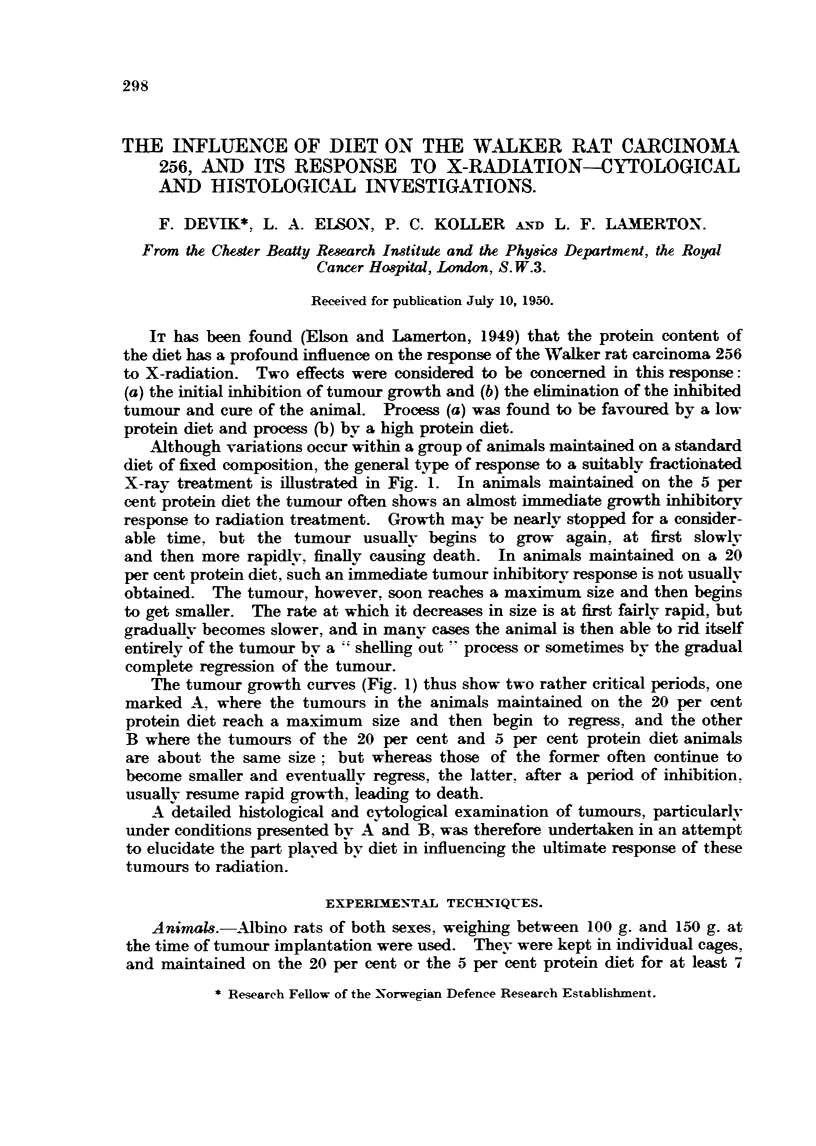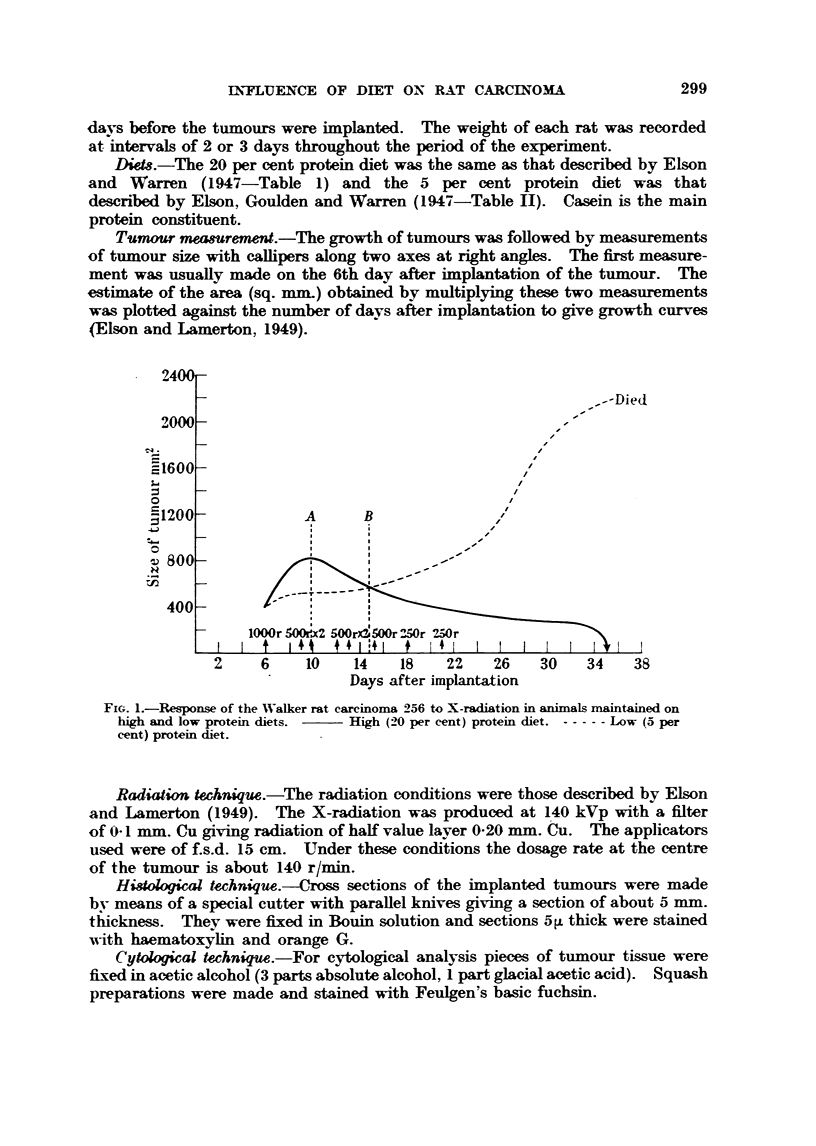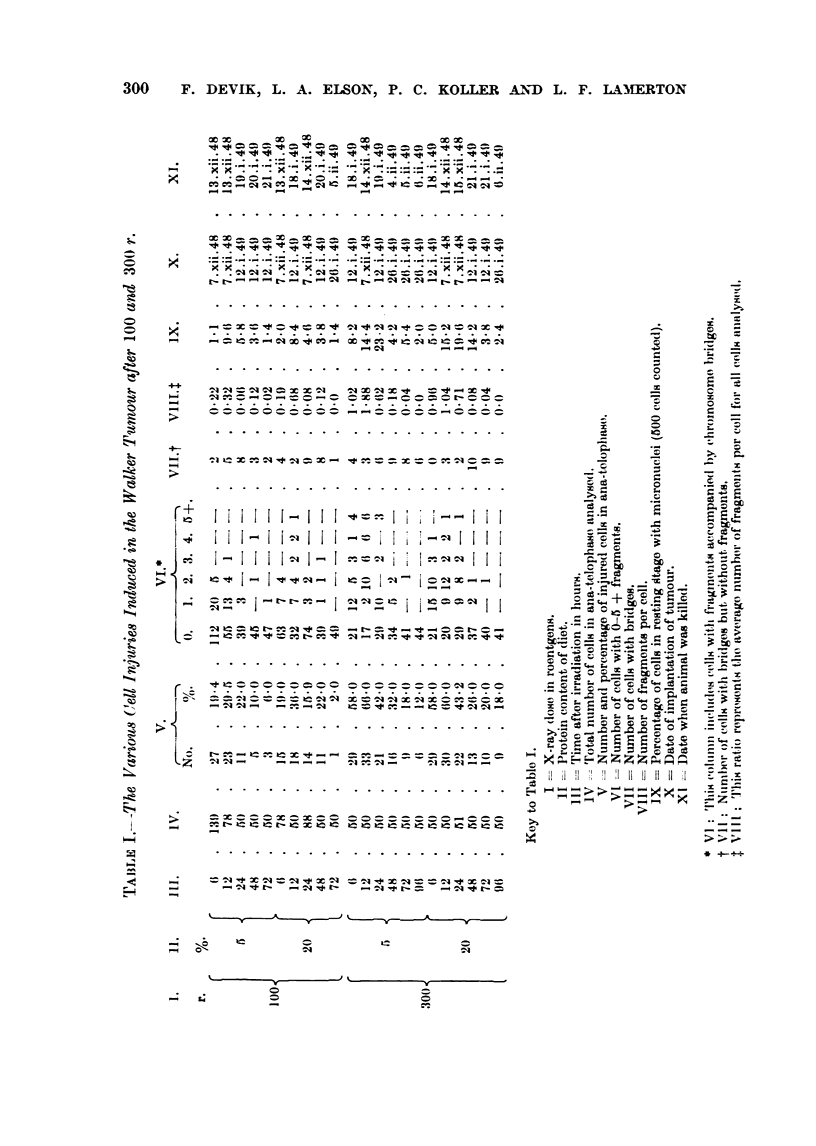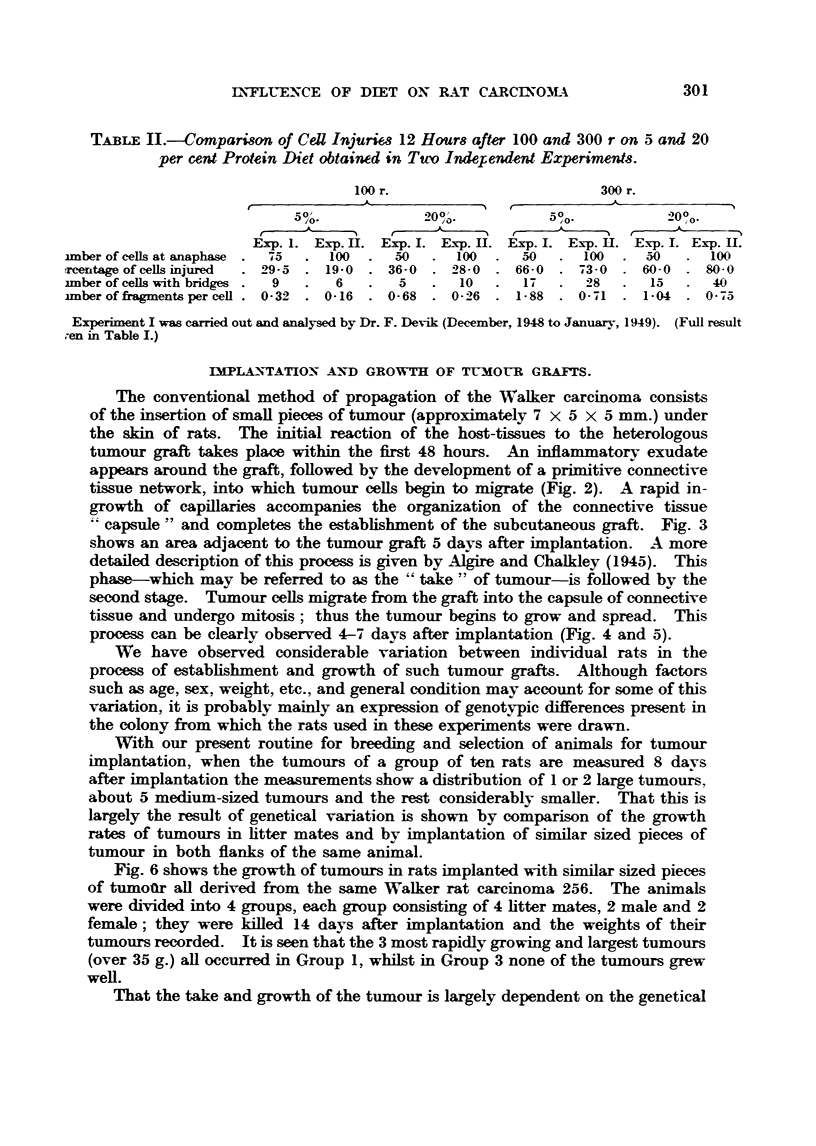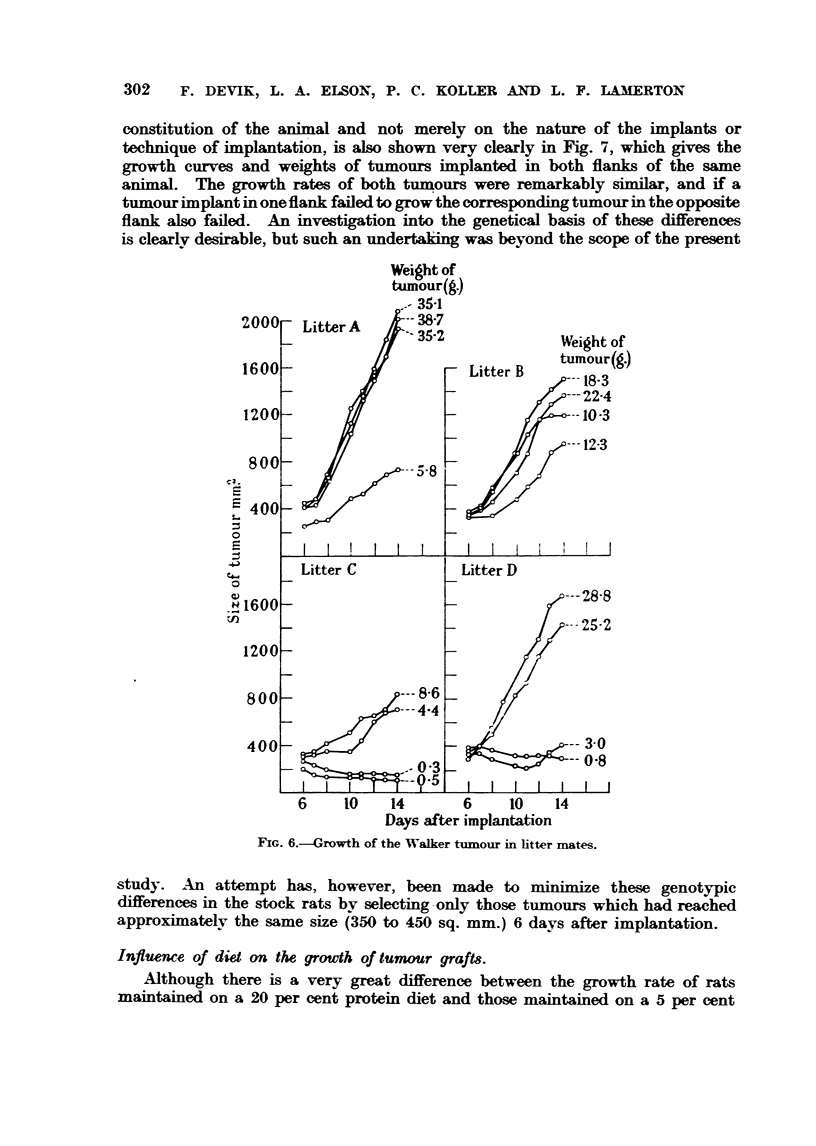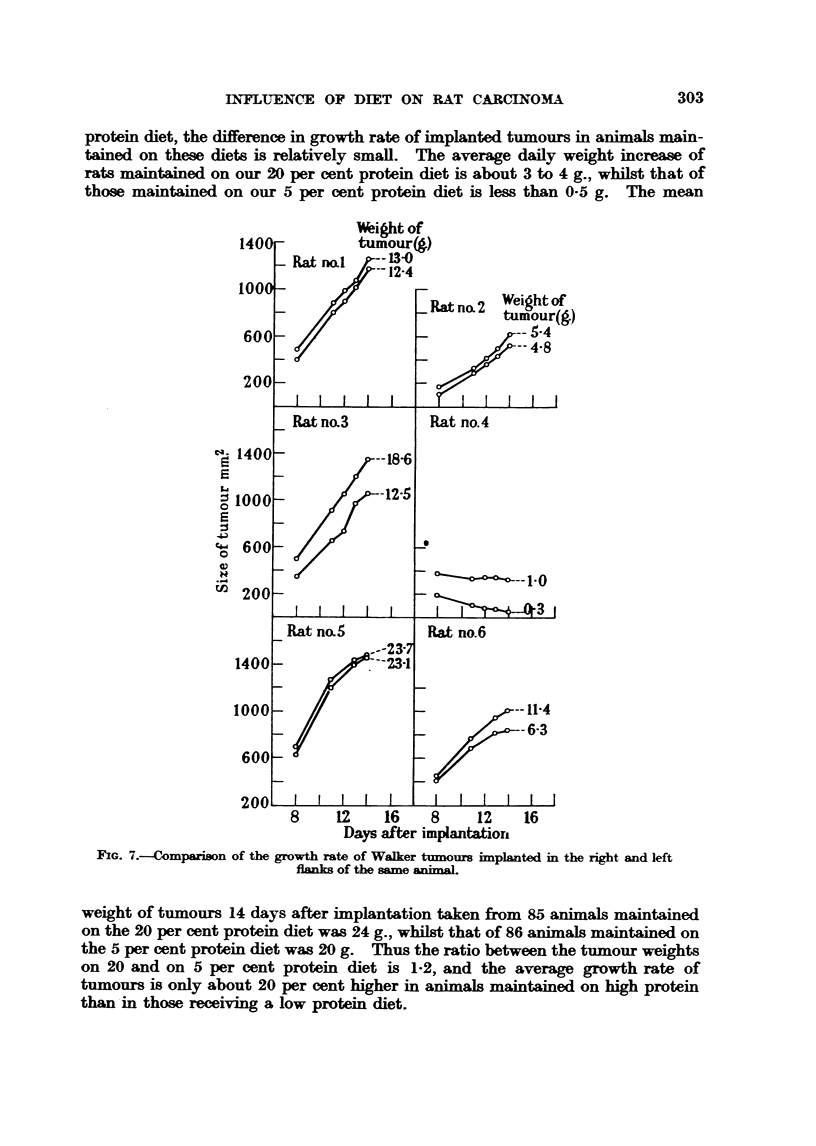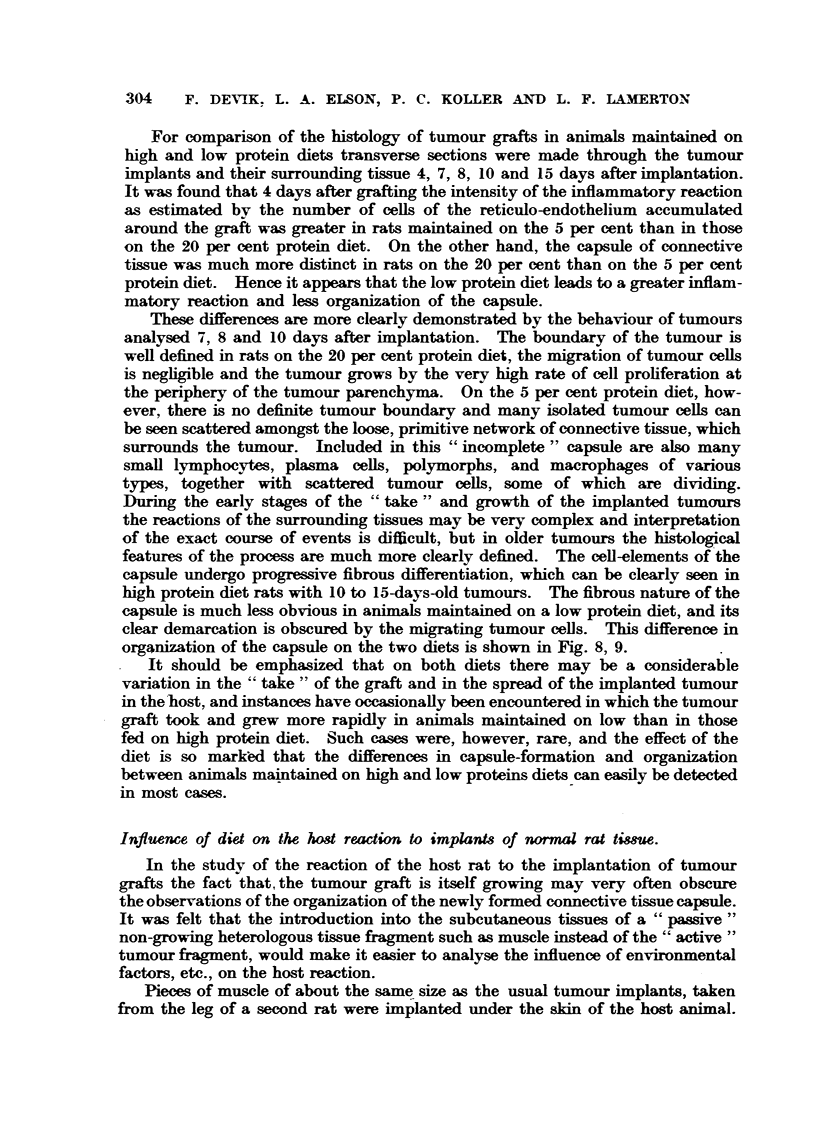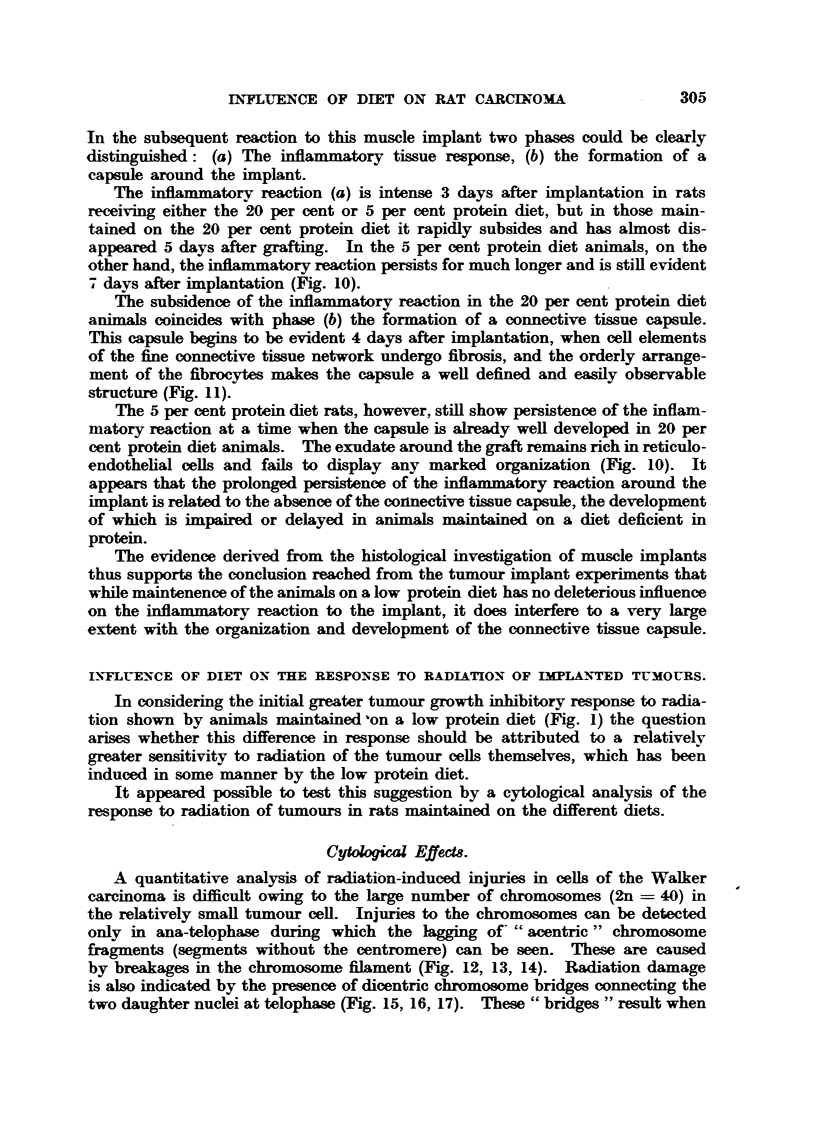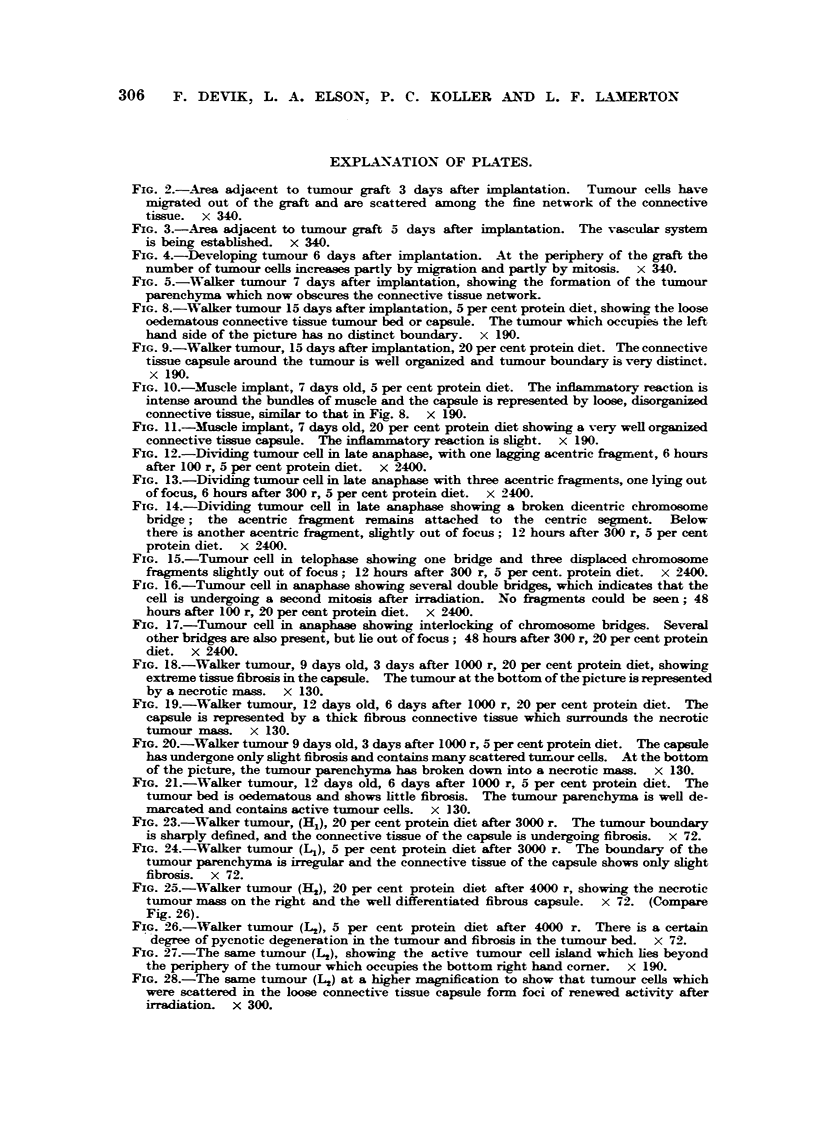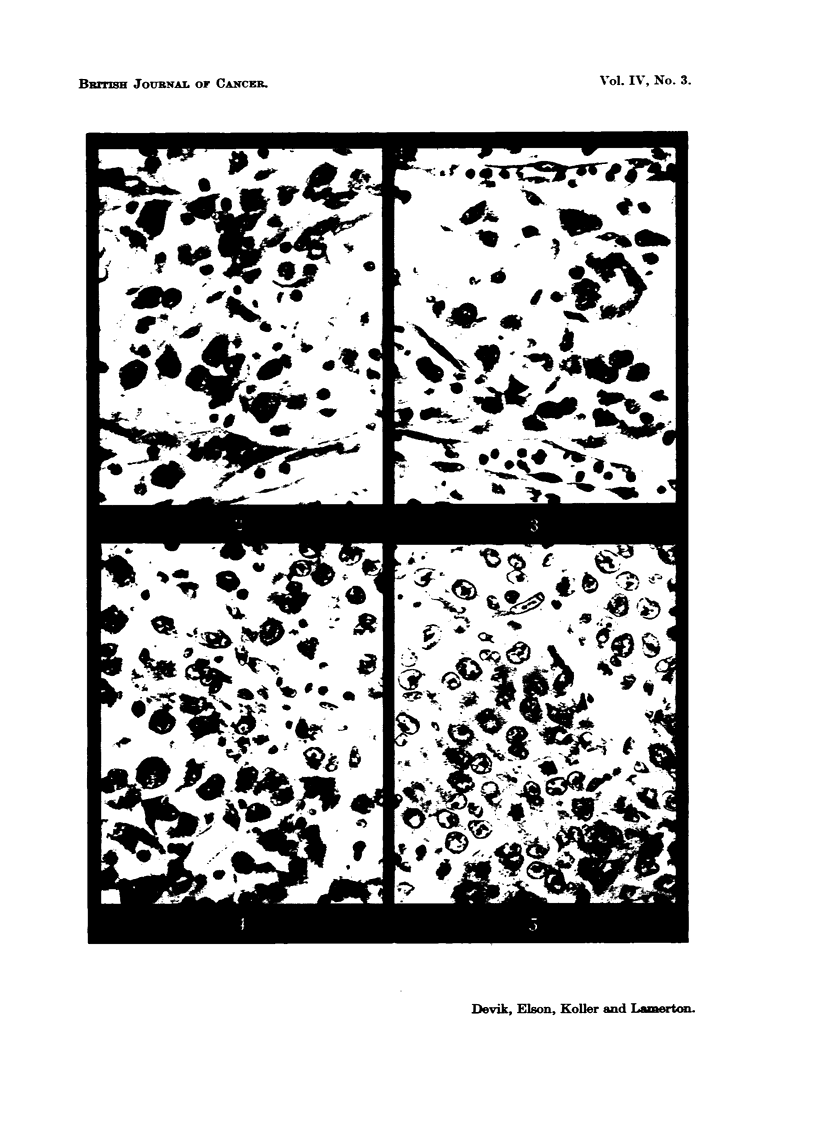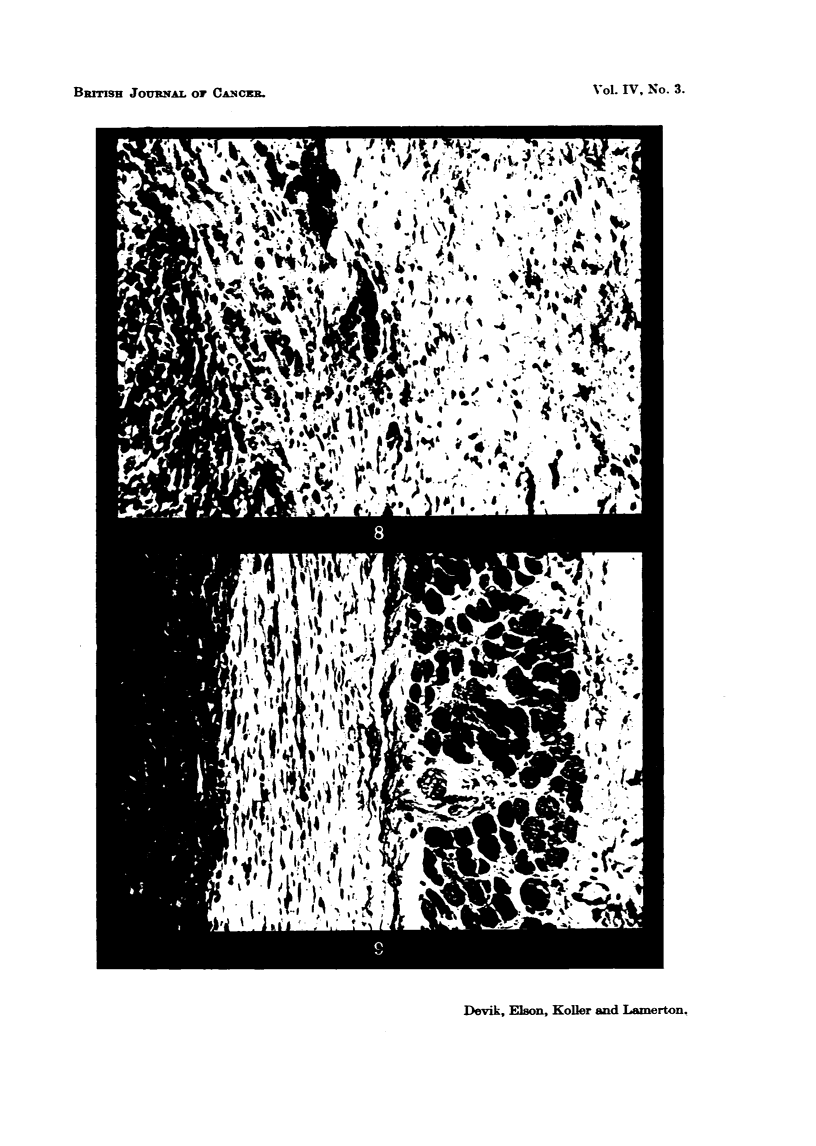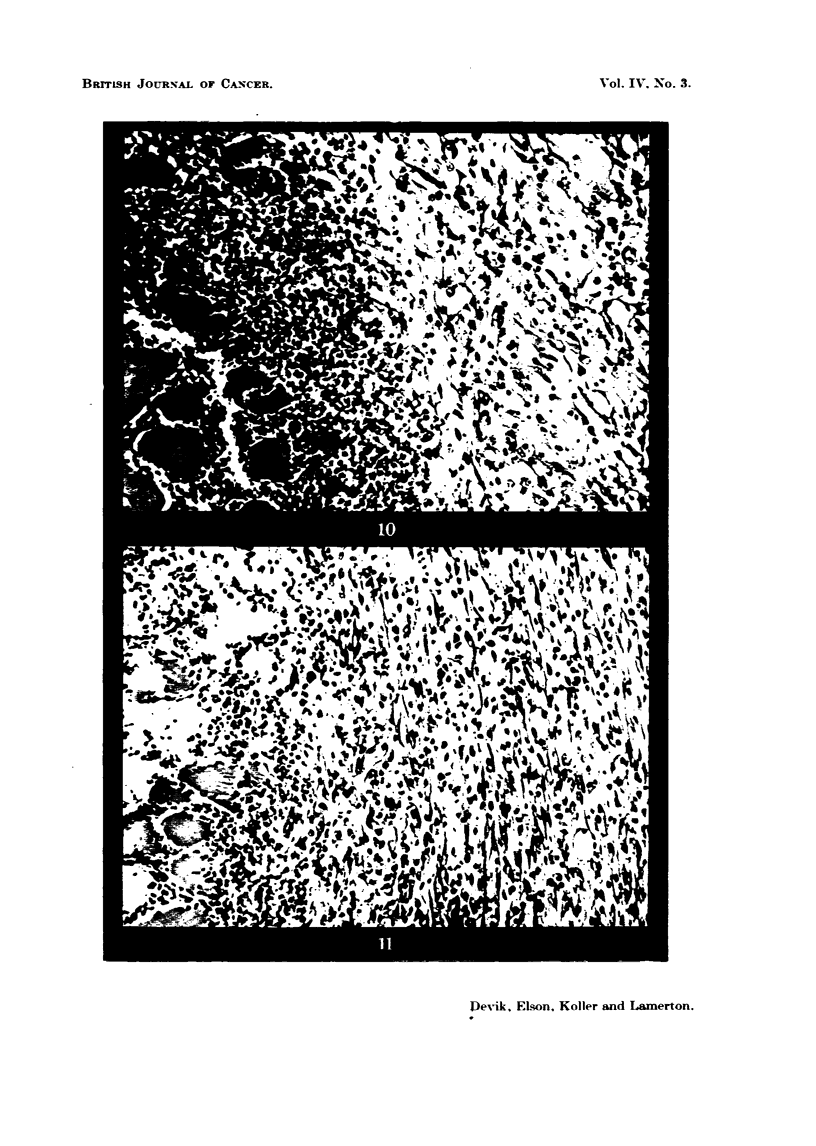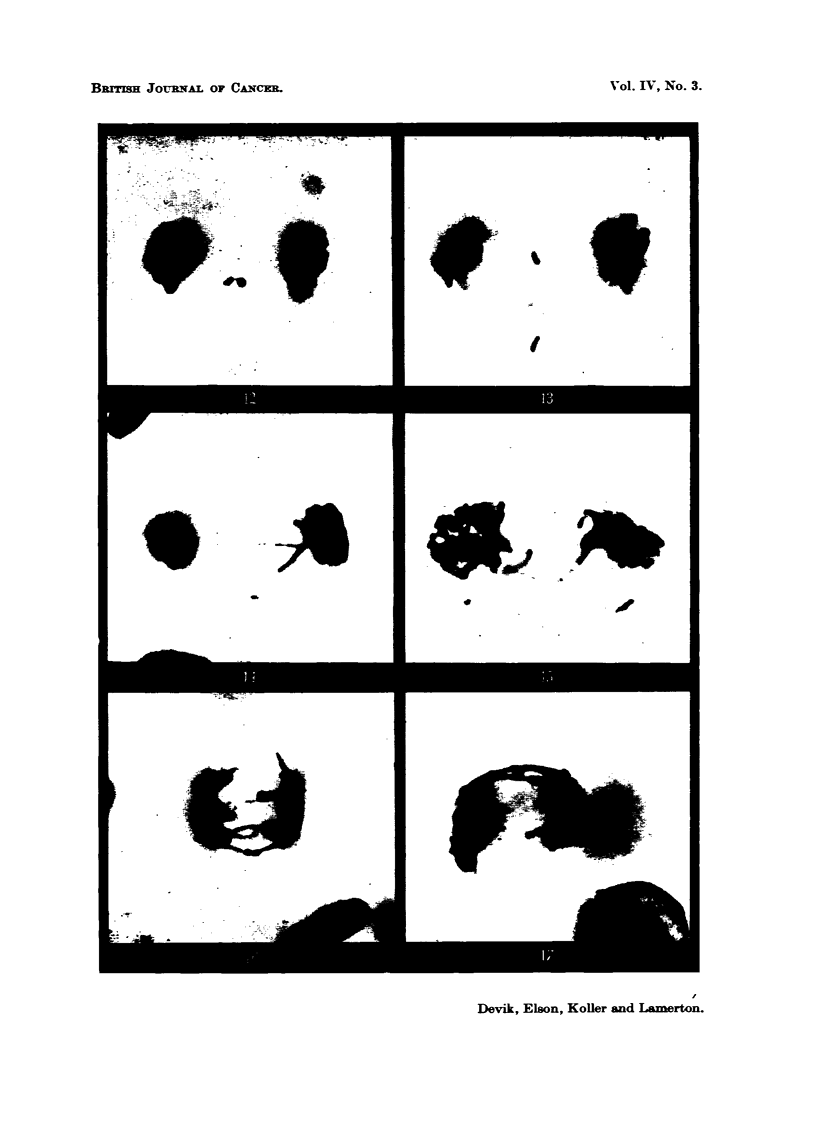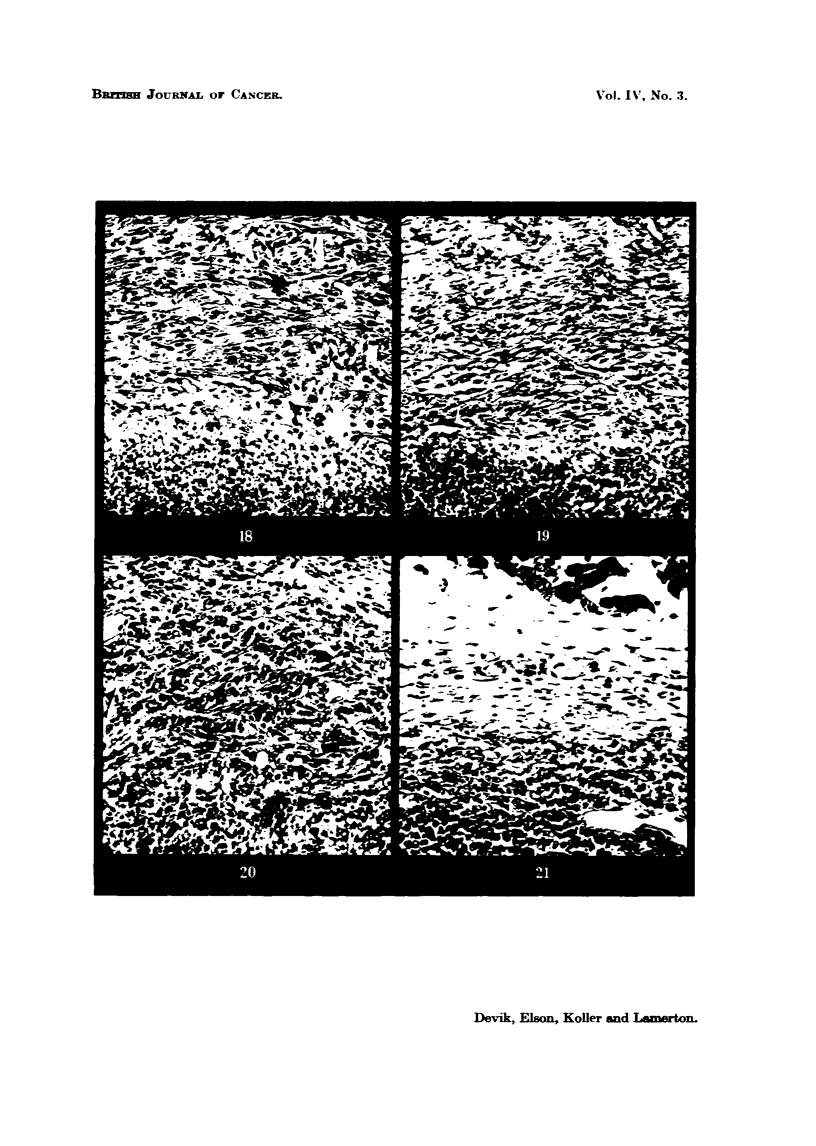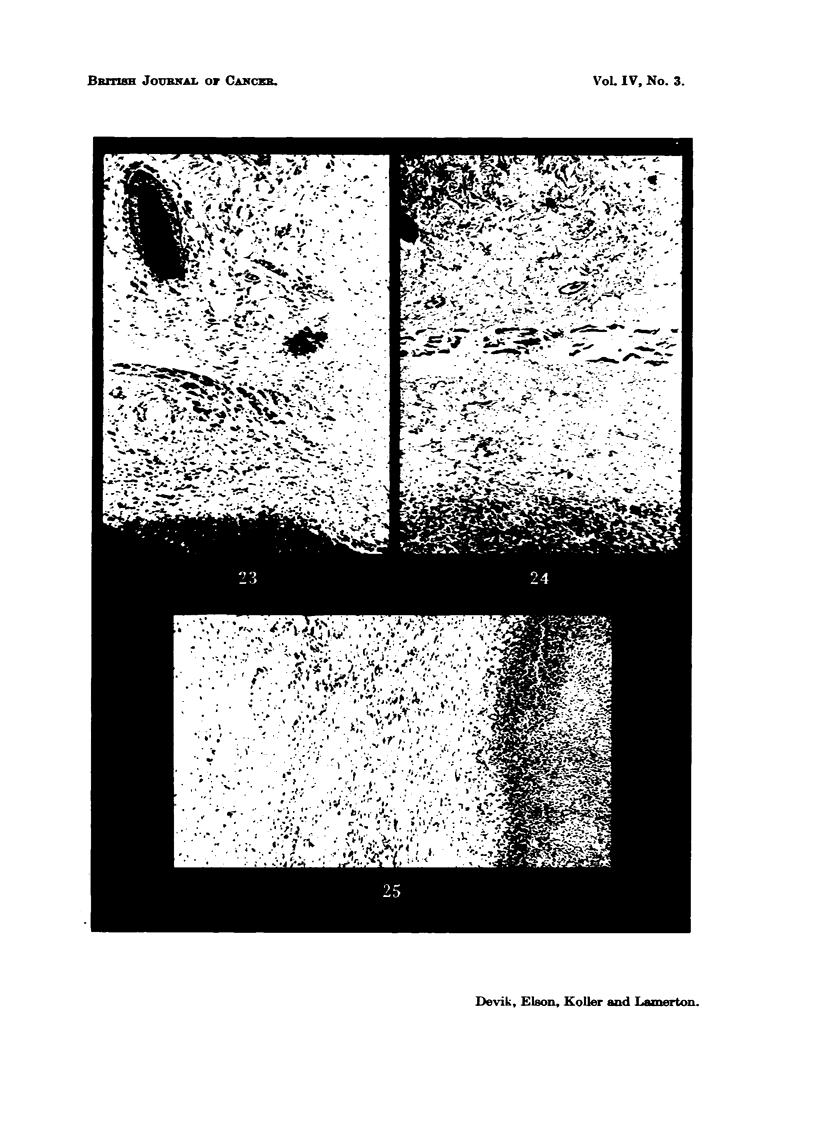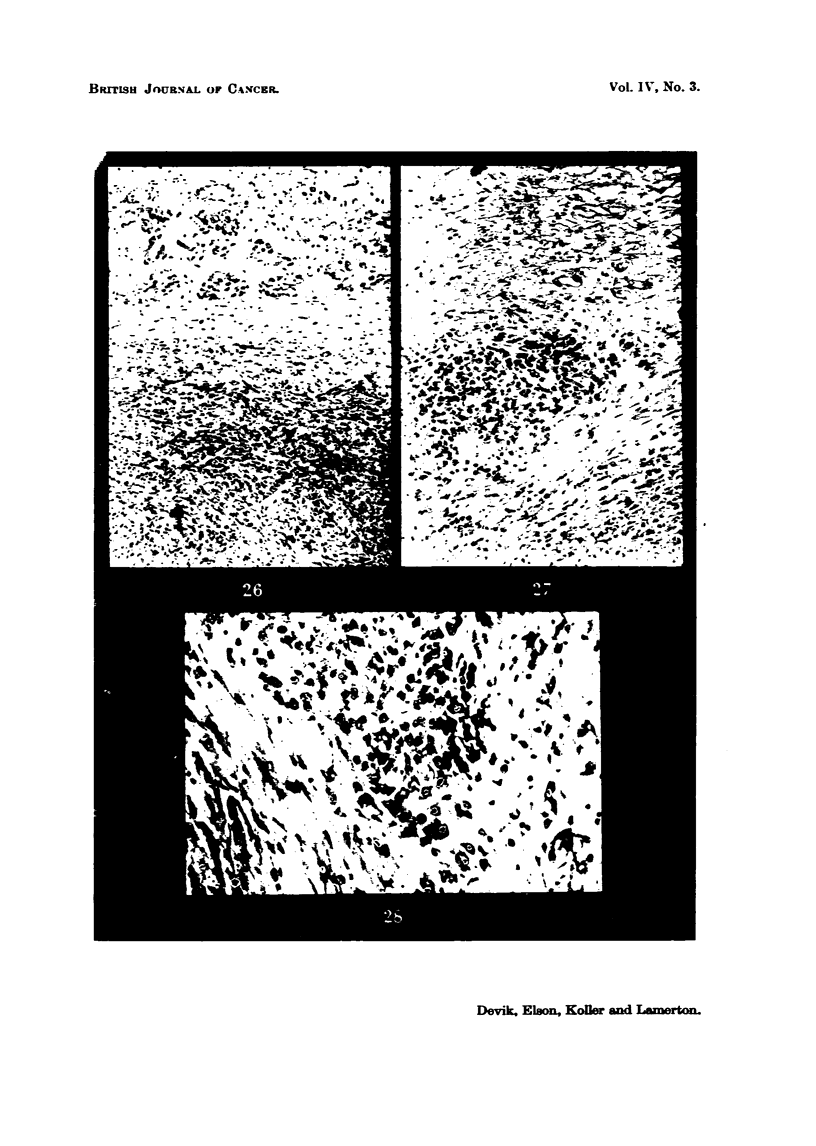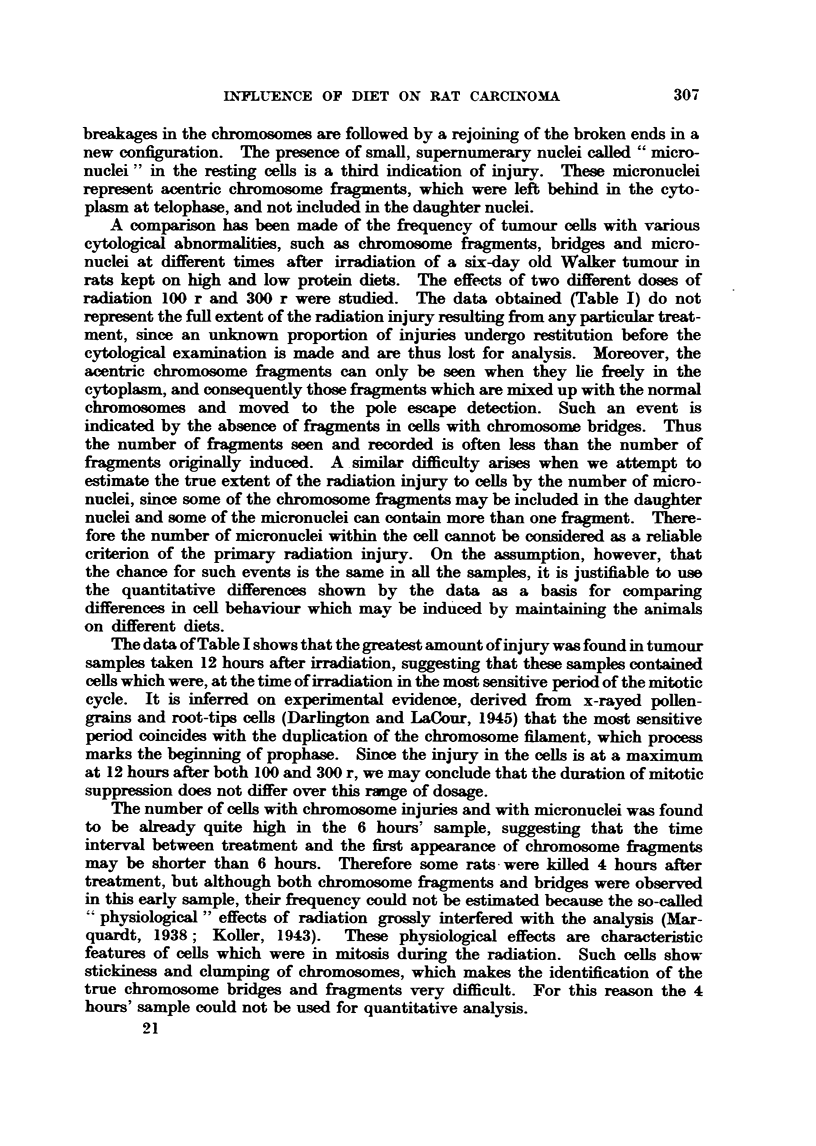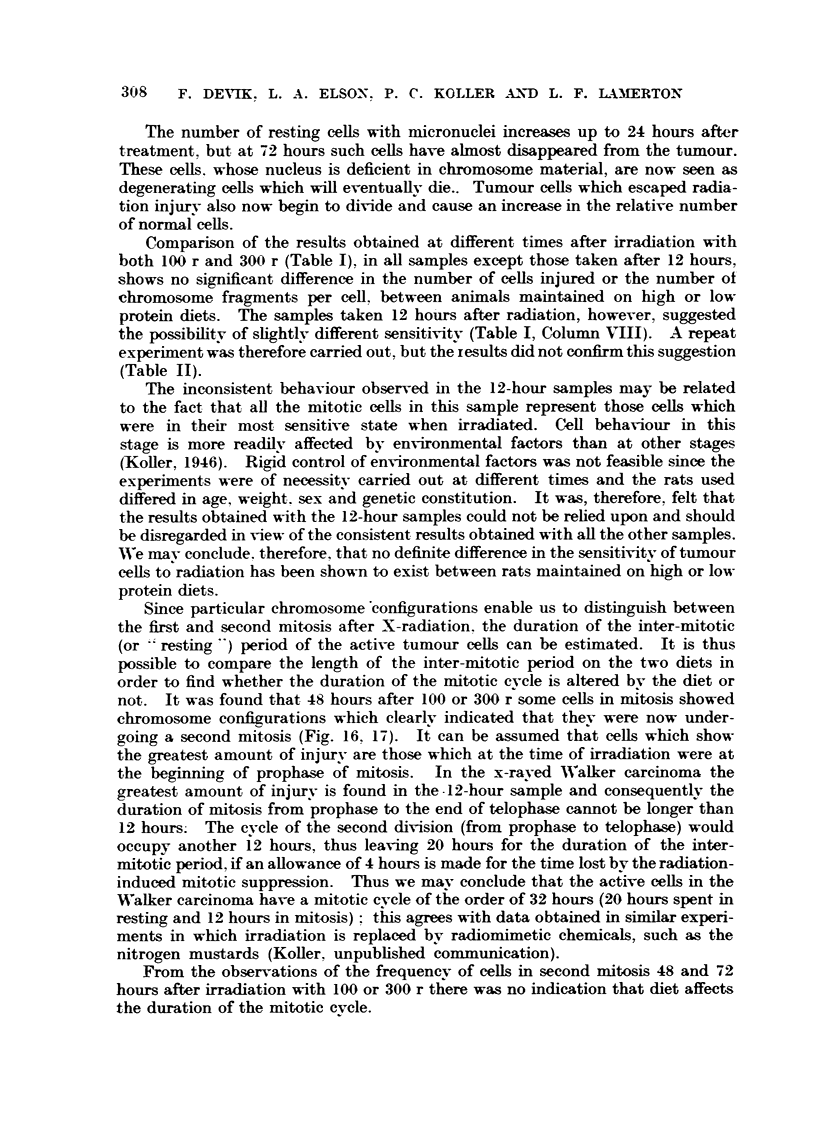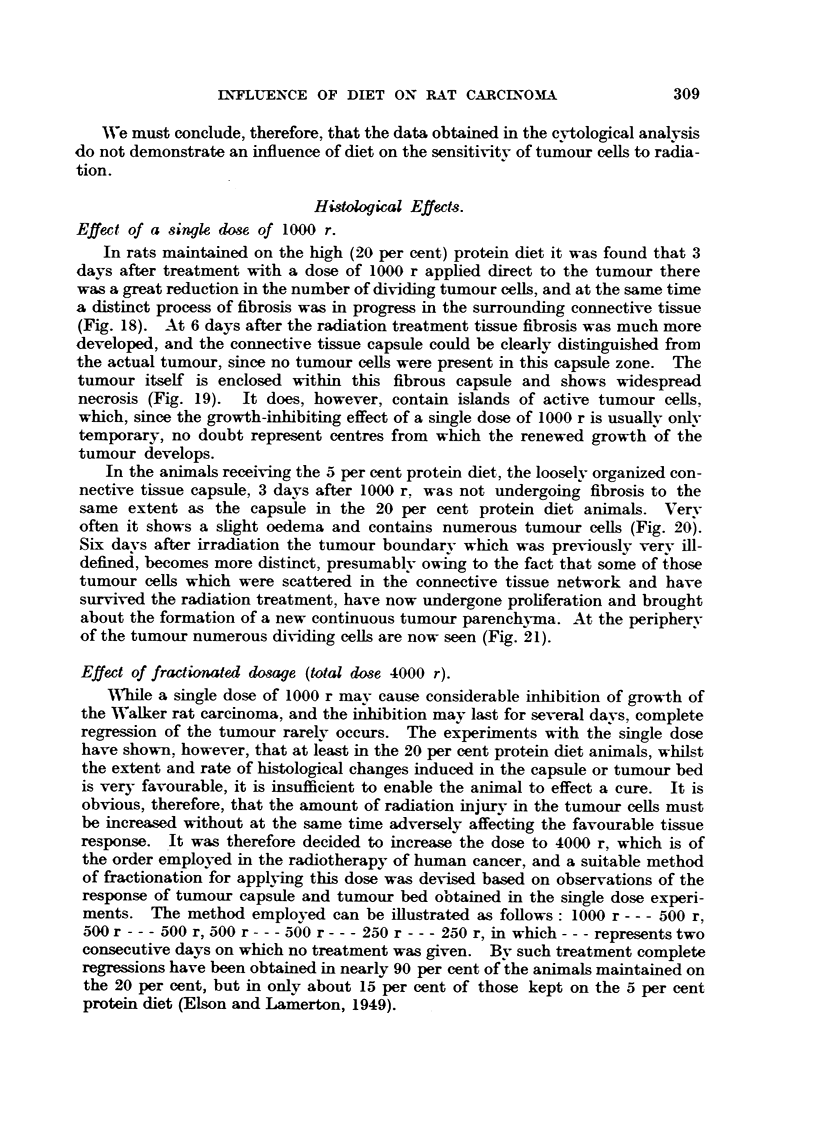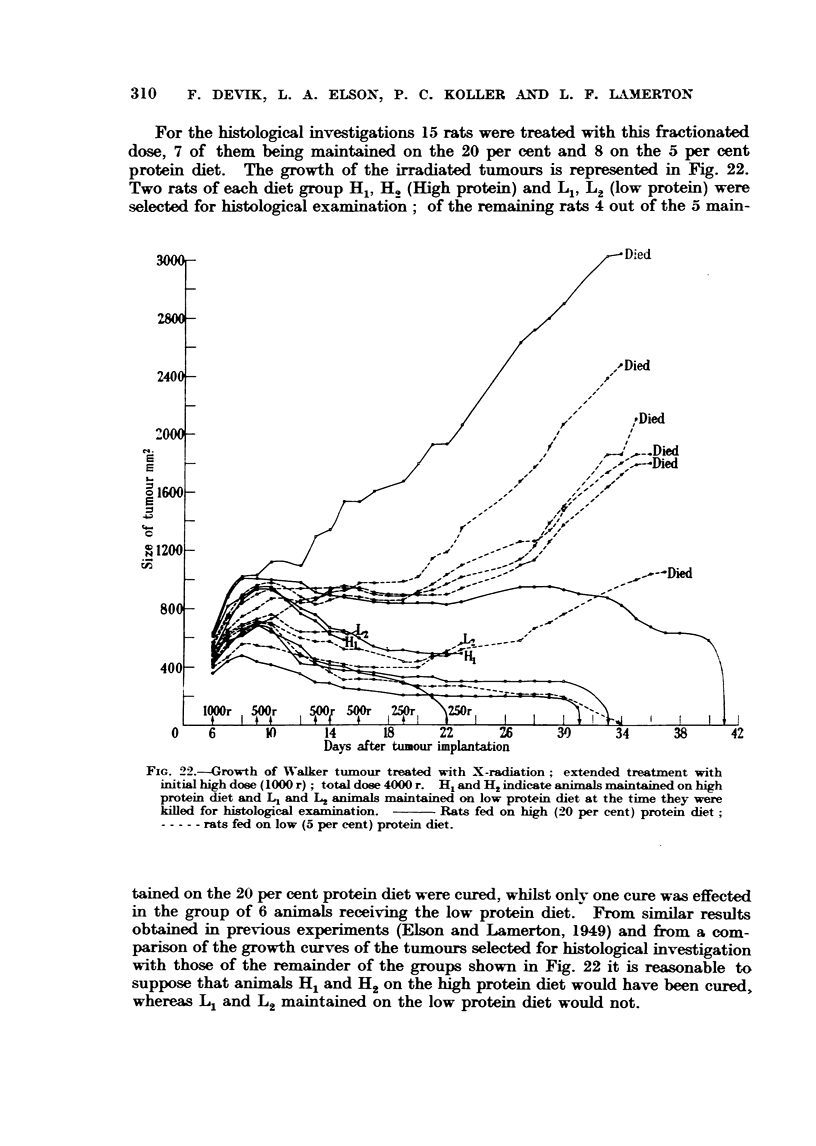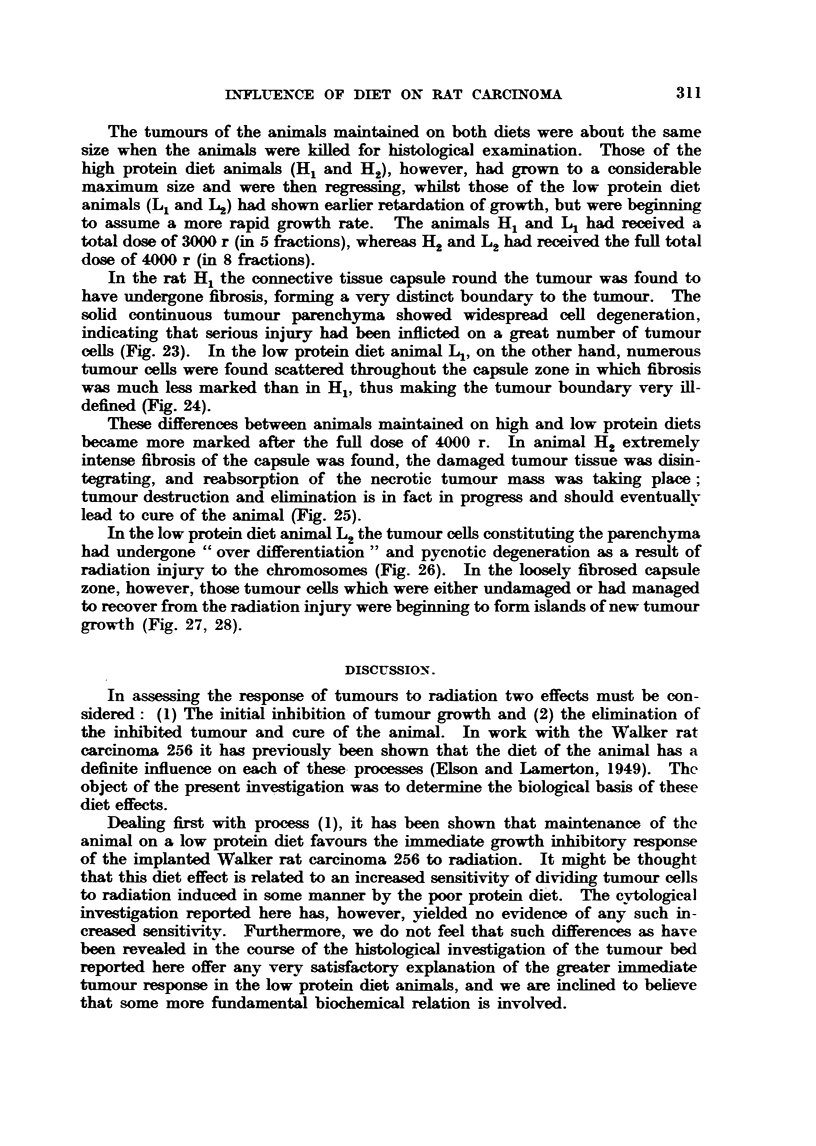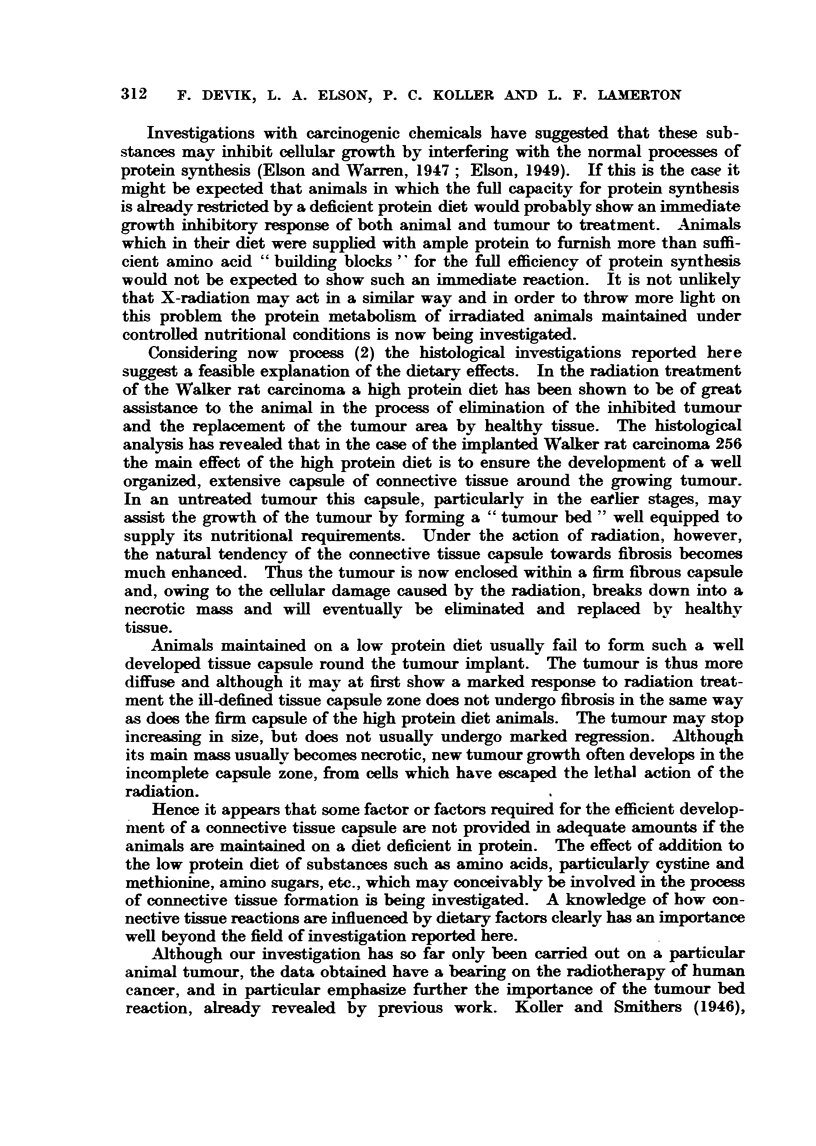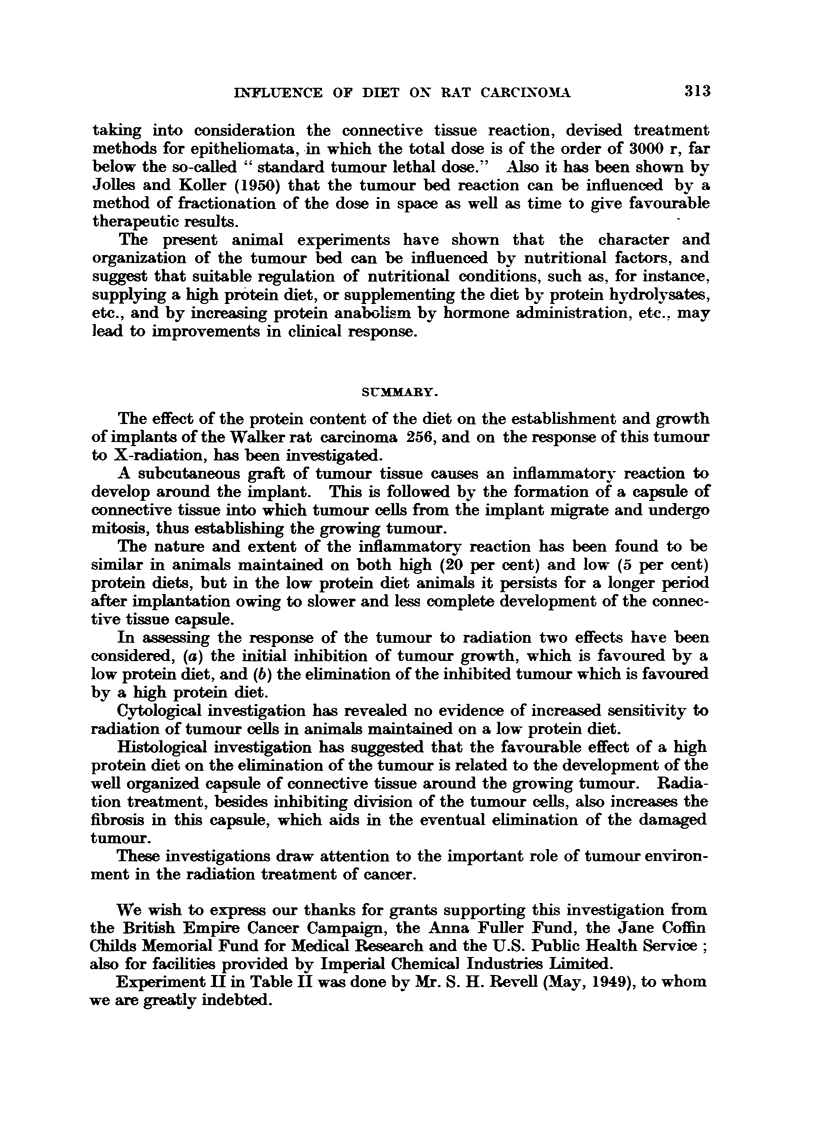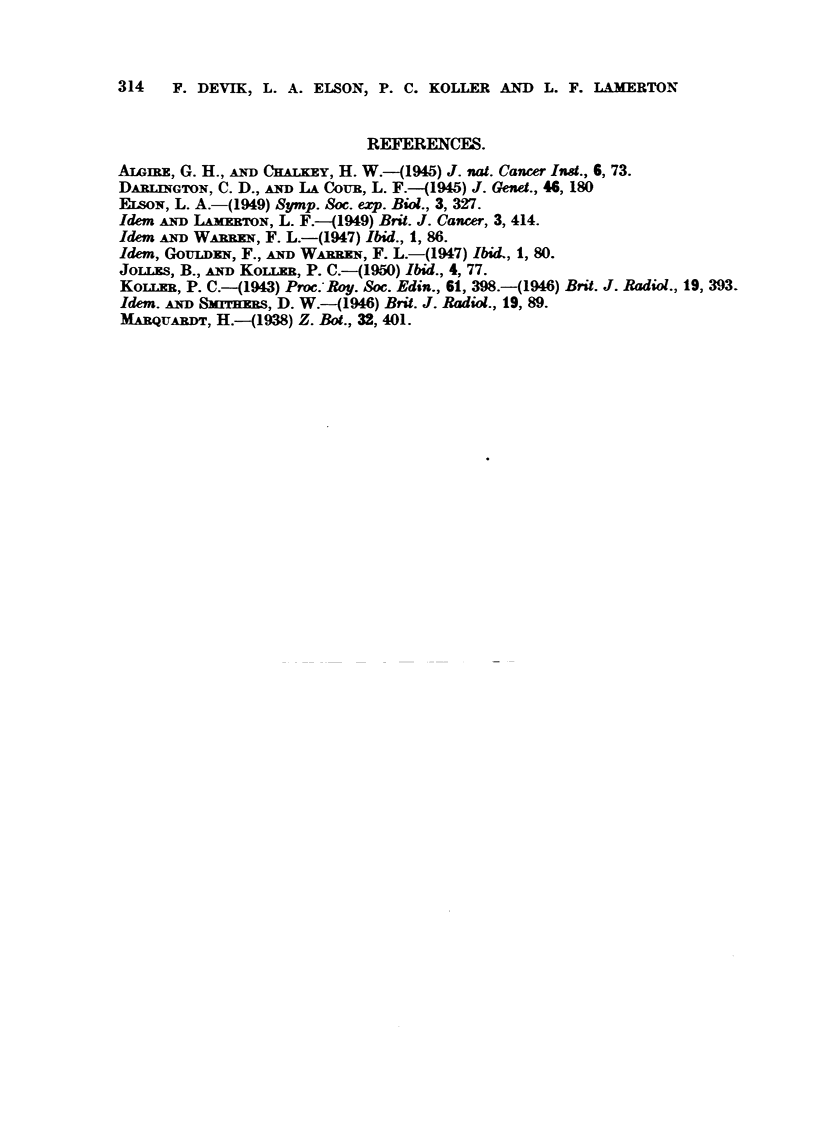# The Influence of Diet on the Walker Rat Carcinoma 256, and Its Response to X-Radiation—Cytological and Histological Investigations

**DOI:** 10.1038/bjc.1950.28

**Published:** 1950-09

**Authors:** F. Devik, L. A. Elson, P. C. Koller, L. F. Lamerton

## Abstract

**Images:**


### THE INFLUENCE OF DIET ON THE WALKER RAT CARCINOMA 256, AND ITS RESPONSE TO X-RADIATION—CYTOLOGICAL AND HISTOLOGICAL INVESTIGATIONS.

F. DEVIK*, L. A. ELSON, P. C. KOLLER AND L. F. LAMERTON.

*From the Chester Beatty Research Institute and the Physics Department, the Royal Cancer Hospital, London, S.W.3.*

Received for publication July 10, 1950.

IT has been found (Elson and Lamerton, 1949) that the protein content of the diet has a profound influence on the response of the Walker rat carcinoma 256 to X-radiation. Two effects were considered to be concerned in this response: (*a*) the initial inhibition of tumour growth and (*b*) the elimination of the inhibited tumour and cure of the animal. Process (*a*) was found to be favoured by a low protein diet and process (b) by a high protein diet.

Although variations occur within a group of animals maintained on a standard diet of fixed composition, the general type of response to a suitably fractionated X-ray treatment is illustrated in Fig. 1. In animals maintained on the 5 per cent protein diet the tumour often shows an almost immediate growth inhibitory response to radiation treatment. Growth may be nearly stopped for a considerable time, but the tumour usually begins to grow again, at first slowly and then more rapidly, finally causing death. In animals maintained on a 20 per cent protein diet, such an immediate tumour inhibitory response is not usually obtained. The tumour, however, soon reaches a maximum size and then begins to get smaller. The rate at which it decreases in size is at first fairly rapid, but gradually becomes slower, and in many cases the animal is then able to rid itself entirely of the tumour by a " shelling out " process or sometimes by the gradual complete regression of the tumour.

The tumour growth curves (Fig. 1) thus show two rather critical periods, one marked A, where the tumours in the animals maintained on the 20 per cent protein diet reach a maximum size and then begin to regress, and the other B where the tumours of the 20 per cent and 5 per cent protein diet animals are about the same size ; but whereas those of the former often continue to become smaller and eventually regress, the latter, after a period of inhibition, usually resume rapid growth, leading to death.

A detailed histological and cytological examination of tumours, particularly under conditions presented by A and B, was therefore undertaken in an attempt to elucidate the part played by diet in influencing the ultimate response of these tumours to radiation.

### EXPERIMENTAL TECHNIQUES.

*Animals.*—Albino rats of both sexes, weighing between 100 g. and 150 g. at the time of tumour implantation were used. They were kept in individual cages, and maintained on the 20 per cent or the 5 per cent protein diet for at least 7

* Research Fellow of the Norwegian Defence Research Establishment.

days before the tumours were implanted. The weight of each rat was recorded at intervals of 2 or 3 days throughout the period of the experiment.

*Diets.*—The 20 per cent protein diet was the same as that described by Elson and Warren (1947—Table 1) and the 5 per cent protein diet was that described by Elson, Goulden and Warren (1947—Table II). Casein is the main protein constituent.

*Tumour measurement.*—The growth of tumours was followed by measurements of tumour size with callipers along two axes at right angles. The first measurement was usually made on the 6th day after implantation of the tumour. The estimate of the area (sq. mm.) obtained by multiplying these two measurements was plotted against the number of days after implantation to give growth curves (Elson and Lamerton, 1949).

FIG. 1.—Response of the Walker rat carcinoma 256 to X-radiation in animals maintained on high and low protein diets. ——— High (20 per cent) protein diet. - - - - - Low (5 per cent) protein diet.

*Radiation technique.*—The radiation conditions were those described by Elson and Lamerton (1949). The X-radiation was produced at 140 kVp with a filter of 0·1 mm. Cu giving radiation of half value layer 0·20 mm. Cu. The applicators used were of f.s.d. 15 cm. Under these conditions the dosage rate at the centre of the tumour is about 140 r/min.

*Histological technique.*—Cross sections of the implanted tumours were made by means of a special cutter with parallel knives giving a section of about 5 mm. thickness. They were fixed in Bouin solution and sections 5$\mu$ thick were stained with haematoxylin and orange G.

*Cytological technique.*—For cytological analysis pieces of tumour tissue were fixed in acetic alcohol (3 parts absolute alcohol, 1 part glacial acetic acid). Squash preparations were made and stained with Feulgen's basic fuchsin.

TABLE I.—*The Various Cell Injuries Induced in the Walker Tumour after* 100 *and* 300 *r.*

| I. | II. | III. | IV. | V. | | VI.* | | | | | | VII.† | VIII.‡ | IX. | X. | XI. |
|---|---|---|---|---|---|---|---|---|---|---|---|---|---|---|---|---|
| r. | %. | | | No. | %. | 0. | 1. | 2. | 3. | 4. | 5+. | | | | | |
| 100 | 5 | 6 | 130 | 27 | 19·4 | 112 | 20 | 5 | — | — | — | 2 | 0·22 | 1·1 | 7.xii.48 | 13.xii.48 |
| | | 12 | 78 | 23 | 29·5 | 55 | 13 | 4 | 1 | — | — | 5 | 0·32 | 9·6 | 7.xii.48 | 13.xii.48 |
| | | 24 | 50 | 11 | 22·0 | 39 | 3 | — | — | — | — | 8 | 0·06 | 5·8 | 12.i.49 | 19.i.49 |
| | | 48 | 50 | 5 | 10·0 | 45 | — | 1 | — | 1 | — | 3 | 0·12 | 3·6 | 12.i.49 | 20.i.49 |
| | | 72 | 50 | 3 | 6·0 | 47 | 1 | — | — | — | — | 2 | 0·02 | 1·4 | 12.i.49 | 21.i.49 |
| | 20 | 6 | 78 | 15 | 19·0 | 63 | 7 | 4 | — | — | — | 4 | 0·19 | 2·0 | 7.xii.48 | 13.xii.48 |
| | | 12 | 50 | 18 | 36·0 | 32 | 7 | 4 | 2 | 2 | 1 | 2 | 0·68 | 8·4 | 12.i.49 | 18.i.49 |
| | | 24 | 88 | 14 | 15·9 | 74 | 3 | 2 | — | — | — | 9 | 0·08 | 4·6 | 7.xii.48 | 14.xii.48 |
| | | 48 | 50 | 11 | 22·0 | 39 | 1 | 1 | 1 | — | — | 8 | 0·12 | 3·8 | 12.i.49 | 20.i.49 |
| | | 72 | 50 | 1 | 2·0 | 49 | — | — | — | — | — | 1 | 0·0 | 1·4 | 26.i.49 | 5.ii.49 |
| 300 | 5 | 6 | 50 | 29 | 58·0 | 21 | 12 | 5 | 3 | 1 | 4 | 4 | 1·02 | 8·2 | 12.i.49 | 18.i.49 |
| | | 12 | 50 | 33 | 66·0 | 17 | 2 | 10 | 6 | 6 | 6 | 3 | 1·88 | 14·4 | 7.xii.48 | 14.xii.48 |
| | | 24 | 50 | 21 | 42·0 | 29 | 10 | — | 2 | — | 3 | 6 | 0·62 | 23·2 | 12.i.49 | 19.i.49 |
| | | 48 | 50 | 16 | 32·0 | 34 | 5 | 2 | — | — | — | 9 | 0·18 | 4·2 | 26.i.49 | 4.ii.49 |
| | | 72 | 50 | 9 | 18·0 | 41 | — | 1 | — | — | — | 8 | 0·04 | 5·4 | 26.i.49 | 5.ii.49 |
| | | 96 | 50 | 6 | 12·0 | 44 | — | — | — | — | — | 6 | 0·0 | 2·0 | 26.i.49 | 6.ii.49 |
| | 20 | 6 | 50 | 29 | 58·0 | 21 | 15 | 10 | 3 | 1 | — | 0 | 0·96 | 5·0 | 12.i.49 | 18.i.49 |
| | | 12 | 50 | 30 | 60·0 | 20 | 9 | 12 | 2 | 2 | 1 | 3 | 1·04 | 15·2 | 7.xii.48 | 14.xii.48 |
| | | 24 | 51 | 22 | 43·2 | 29 | 9 | 8 | 2 | — | 1 | 2 | 0·71 | 19·6 | 7.xii.48 | 15.xii.48 |
| | | 48 | 50 | 13 | 26·0 | 37 | 2 | 1 | — | — | — | 10 | 0·08 | 14·2 | 12.i.49 | 21.i.49 |
| | | 72 | 50 | 10 | 20·0 | 40 | — | 1 | — | — | — | 9 | 0·04 | 3·8 | 12.i.49 | 21.i.49 |
| | | 96 | 50 | 9 | 18·0 | 41 | — | — | — | — | — | 9 | 0·0 | 2·4 | 26.i.49 | 6.ii.49 |

Key to Table I.

I = X-ray dose in roentgens.

II = Protein content of diet.

III = Time after irradiation in hours.

IV = Total number of cells in ana-telophase analysed.

V = Number and percentage of injured cells in ana-telophase.

VI = Number of cells with 0–5 + fragments.

VII = Number of cells with bridges.

VIII = Number of fragments per cell.

IX = Percentage of cells in resting stage with micronuclei (500 cells counted).

X = Date of implantation of tumour.

XI = Date when animal was killed.

* VI : This column includes cells with fragments accompanied by chromosome bridges.

† VII : Number of cells with bridges but without fragments.

‡ VIII : This ratio represents the average number of fragments per cell for all cells analysed.

TABLE II.—*Comparison of Cell Injuries* 12 *Hours after* 100 *and* 300 *r on* 5 *and* 20 *per cent Protein Diet obtained in Two Independent Experiments.*

| | 100 r. | | | | 300 r. | | | |
|---|---|---|---|---|---|---|---|---|
| | 5%. | | 20%. | | 5%. | | 20%. | |
| | Exp. I. | Exp. II. | Exp. I. | Exp. II. | Exp. I. | Exp. II. | Exp. I. | Exp. II. |
| umber of cells at anaphase | 75 | 100 | 50 | 100 | 50 | 100 | 50 | 100 |
| rcentage of cells injured | 29·5 | 19·0 | 36·0 | 28·0 | 66·0 | 73·0 | 60·0 | 80·0 |
| umber of cells with bridges | 9 | 6 | 5 | 10 | 17 | 28 | 15 | 40 |
| umber of fragments per cell | 0·32 | 0·16 | 0·68 | 0·26 | 1·88 | 0·71 | 1·04 | 0·75 |

Experiment I was carried out and analysed by Dr. F. Devik (December, 1948 to January, 1949). (Full result ven in Table I.)

### IMPLANTATION AND GROWTH OF TUMOUR GRAFTS.

The conventional method of propagation of the Walker carcinoma consists of the insertion of small pieces of tumour (approximately 7 × 5 × 5 mm.) under the skin of rats. The initial reaction of the host-tissues to the heterologous tumour graft takes place within the first 48 hours. An inflammatory exudate appears around the graft, followed by the development of a primitive connective tissue network, into which tumour cells begin to migrate (Fig. 2). A rapid ingrowth of capillaries accompanies the organization of the connective tissue " capsule " and completes the establishment of the subcutaneous graft. Fig. 3 shows an area adjacent to the tumour graft 5 days after implantation. A more detailed description of this process is given by Algire and Chalkley (1945). This phase—which may be referred to as the " take " of tumour—is followed by the second stage. Tumour cells migrate from the graft into the capsule of connective tissue and undergo mitosis ; thus the tumour begins to grow and spread. This process can be clearly observed 4–7 days after implantation (Fig. 4 and 5).

We have observed considerable variation between individual rats in the process of establishment and growth of such tumour grafts. Although factors such as age, sex, weight, etc., and general condition may account for some of this variation, it is probably mainly an expression of genotypic differences present in the colony from which the rats used in these experiments were drawn.

With our present routine for breeding and selection of animals for tumour implantation, when the tumours of a group of ten rats are measured 8 days after implantation the measurements show a distribution of 1 or 2 large tumours, about 5 medium-sized tumours and the rest considerably smaller. That this is largely the result of genetical variation is shown by comparison of the growth rates of tumours in litter mates and by implantation of similar sized pieces of tumour in both flanks of the same animal.

Fig. 6 shows the growth of tumours in rats implanted with similar sized pieces of tumour all derived from the same Walker rat carcinoma 256. The animals were divided into 4 groups, each group consisting of 4 litter mates, 2 male and 2 female ; they were killed 14 days after implantation and the weights of their tumours recorded. It is seen that the 3 most rapidly growing and largest tumours (over 35 g.) all occurred in Group 1, whilst in Group 3 none of the tumours grew well.

That the take and growth of the tumour is largely dependent on the genetical constitution of the animal and not merely on the nature of the implants or technique of implantation, is also shown very clearly in Fig. 7, which gives the growth curves and weights of tumours implanted in both flanks of the same animal. The growth rates of both tumours were remarkably similar, and if a tumour implant in one flank failed to grow the corresponding tumour in the opposite flank also failed. An investigation into the genetical basis of these differences is clearly desirable, but such an undertaking was beyond the scope of the present

FIG. 6.—Growth of the Walker tumour in litter mates.

study. An attempt has, however, been made to minimize these genotypic differences in the stock rats by selecting only those tumours which had reached approximately the same size (350 to 450 sq. mm.) 6 days after implantation.

*Influence of diet on the growth of tumour grafts.*

Although there is a very great difference between the growth rate of rats maintained on a 20 per cent protein diet and those maintained on a 5 per cent protein diet, the difference in growth rate of implanted tumours in animals maintained on these diets is relatively small. The average daily weight increase of rats maintained on our 20 per cent protein diet is about 3 to 4 g., whilst that of those maintained on our 5 per cent protein diet is less than 0·5 g. The mean

FIG. 7.—Comparison of the growth rate of Walker tumours implanted in the right and left flanks of the same animal.

weight of tumours 14 days after implantation taken from 85 animals maintained on the 20 per cent protein diet was 24 g., whilst that of 86 animals maintained on the 5 per cent protein diet was 20 g. Thus the ratio between the tumour weights on 20 and on 5 per cent protein diet is 1·2, and the average growth rate of tumours is only about 20 per cent higher in animals maintained on high protein than in those receiving a low protein diet.

For comparison of the histology of tumour grafts in animals maintained on high and low protein diets transverse sections were made through the tumour implants and their surrounding tissue 4, 7, 8, 10 and 15 days after implantation. It was found that 4 days after grafting the intensity of the inflammatory reaction as estimated by the number of cells of the reticulo-endothelium accumulated around the graft was greater in rats maintained on the 5 per cent than in those on the 20 per cent protein diet. On the other hand, the capsule of connective tissue was much more distinct in rats on the 20 per cent than on the 5 per cent protein diet. Hence it appears that the low protein diet leads to a greater inflammatory reaction and less organization of the capsule.

These differences are more clearly demonstrated by the behaviour of tumours analysed 7, 8 and 10 days after implantation. The boundary of the tumour is well defined in rats on the 20 per cent protein diet, the migration of tumour cells is negligible and the tumour grows by the very high rate of cell proliferation at the periphery of the tumour parenchyma. On the 5 per cent protein diet, however, there is no definite tumour boundary and many isolated tumour cells can be seen scattered amongst the loose, primitive network of connective tissue, which surrounds the tumour. Included in this " incomplete " capsule are also many small lymphocytes, plasma cells, polymorphs, and macrophages of various types, together with scattered tumour cells, some of which are dividing. During the early stages of the " take " and growth of the implanted tumours the reactions of the surrounding tissues may be very complex and interpretation of the exact course of events is difficult, but in older tumours the histological features of the process are much more clearly defined. The cell-elements of the capsule undergo progressive fibrous differentiation, which can be clearly seen in high protein diet rats with 10 to 15-days-old tumours. The fibrous nature of the capsule is much less obvious in animals maintained on a low protein diet, and its clear demarcation is obscured by the migrating tumour cells. This difference in organization of the capsule on the two diets is shown in Fig. 8, 9.

It should be emphasized that on both diets there may be a considerable variation in the " take " of the graft and in the spread of the implanted tumour in the host, and instances have occasionally been encountered in which the tumour graft took and grew more rapidly in animals maintained on low than in those fed on high protein diet. Such cases were, however, rare, and the effect of the diet is so marked that the differences in capsule-formation and organization between animals maintained on high and low proteins diets can easily be detected in most cases.

*Influence of diet on the host reaction to implants of normal rat tissue.*

In the study of the reaction of the host rat to the implantation of tumour grafts the fact that the tumour graft is itself growing may very often obscure the observations of the organization of the newly formed connective tissue capsule. It was felt that the introduction into the subcutaneous tissues of a " passive " non-growing heterologous tissue fragment such as muscle instead of the " active " tumour fragment, would make it easier to analyse the influence of environmental factors, etc., on the host reaction.

Pieces of muscle of about the same size as the usual tumour implants, taken from the leg of a second rat were implanted under the skin of the host animal.

In the subsequent reaction to this muscle implant two phases could be clearly distinguished: (*a*) The inflammatory tissue response, (*b*) the formation of a capsule around the implant.

The inflammatory reaction (*a*) is intense 3 days after implantation in rats receiving either the 20 per cent or 5 per cent protein diet, but in those maintained on the 20 per cent protein diet it rapidly subsides and has almost disappeared 5 days after grafting. In the 5 per cent protein diet animals, on the other hand, the inflammatory reaction persists for much longer and is still evident 7 days after implantation (Fig. 10).

The subsidence of the inflammatory reaction in the 20 per cent protein diet animals coincides with phase (*b*) the formation of a connective tissue capsule. This capsule begins to be evident 4 days after implantation, when cell elements of the fine connective tissue network undergo fibrosis, and the orderly arrangement of the fibrocytes makes the capsule a well defined and easily observable structure (Fig. 11).

The 5 per cent protein diet rats, however, still show persistence of the inflammatory reaction at a time when the capsule is already well developed in 20 per cent protein diet animals. The exudate around the graft remains rich in reticulo-endothelial cells and fails to display any marked organization (Fig. 10). It appears that the prolonged persistence of the inflammatory reaction around the implant is related to the absence of the connective tissue capsule, the development of which is impaired or delayed in animals maintained on a diet deficient in protein.

The evidence derived from the histological investigation of muscle implants thus supports the conclusion reached from the tumour implant experiments that while maintenence of the animals on a low protein diet has no deleterious influence on the inflammatory reaction to the implant, it does interfere to a very large extent with the organization and development of the connective tissue capsule.

### INFLUENCE OF DIET ON THE RESPONSE TO RADIATION OF IMPLANTED TUMOURS.

In considering the initial greater tumour growth inhibitory response to radiation shown by animals maintained on a low protein diet (Fig. 1) the question arises whether this difference in response should be attributed to a relatively greater sensitivity to radiation of the tumour cells themselves, which has been induced in some manner by the low protein diet.

It appeared possible to test this suggestion by a cytological analysis of the response to radiation of tumours in rats maintained on the different diets.

### *Cytological Effects.*

A quantitative analysis of radiation-induced injuries in cells of the Walker carcinoma is difficult owing to the large number of chromosomes (2n = 40) in the relatively small tumour cell. Injuries to the chromosomes can be detected only in ana-telophase during which the lagging of " acentric " chromosome fragments (segments without the centromere) can be seen. These are caused by breakages in the chromosome filament (Fig. 12, 13, 14). Radiation damage is also indicated by the presence of dicentric chromosome bridges connecting the two daughter nuclei at telophase (Fig. 15, 16, 17). These " bridges " result when

### EXPLANATION OF PLATES.

FIG. 2.—Area adjacent to tumour graft 3 days after implantation. Tumour cells have migrated out of the graft and are scattered among the fine network of the connective tissue. × 340.

FIG. 3.—Area adjacent to tumour graft 5 days after implantation. The vascular system is being established. × 340.

FIG. 4.—Developing tumour 6 days after implantation. At the periphery of the graft the number of tumour cells increases partly by migration and partly by mitosis. × 340.

FIG. 5.—Walker tumour 7 days after implantation, showing the formation of the tumour parenchyma which now obscures the connective tissue network.

FIG. 8.—Walker tumour 15 days after implantation, 5 per cent protein diet, showing the loose oedematous connective tissue tumour bed or capsule. The tumour which occupies the left hand side of the picture has no distinct boundary. × 190.

FIG. 9.—Walker tumour, 15 days after implantation, 20 per cent protein diet. The connective tissue capsule around the tumour is well organized and tumour boundary is very distinct. × 190.

FIG. 10.—Muscle implant, 7 days old, 5 per cent protein diet. The inflammatory reaction is intense around the bundles of muscle and the capsule is represented by loose, disorganized connective tissue, similar to that in Fig. 8. × 190.

FIG. 11.—Muscle implant, 7 days old, 20 per cent protein diet showing a very well organized connective tissue capsule. The inflammatory reaction is slight. × 190.

FIG. 12.—Dividing tumour cell in late anaphase, with one lagging acentric fragment, 6 hours after 100 r, 5 per cent protein diet. × 2400.

FIG. 13.—Dividing tumour cell in late anaphase with three acentric fragments, one lying out of focus, 6 hours after 300 r, 5 per cent protein diet. × 2400.

FIG. 14.—Dividing tumour cell in late anaphase showing a broken dicentric chromosome bridge; the acentric fragment remains attached to the centric segment. Below there is another acentric fragment, slightly out of focus; 12 hours after 300 r, 5 per cent protein diet. × 2400.

FIG. 15.—Tumour cell in telophase showing one bridge and three displaced chromosome fragments slightly out of focus; 12 hours after 300 r, 5 per cent. protein diet. × 2400.

FIG. 16.—Tumour cell in anaphase showing several double bridges, which indicates that the cell is undergoing a second mitosis after irradiation. No fragments could be seen; 48 hours after 100 r, 20 per cent protein diet. × 2400.

FIG. 17.—Tumour cell in anaphase showing interlocking of chromosome bridges. Several other bridges are also present, but lie out of focus; 48 hours after 300 r, 20 per cent protein diet. × 2400.

FIG. 18.—Walker tumour, 9 days old, 3 days after 1000 r, 20 per cent protein diet, showing extreme tissue fibrosis in the capsule. The tumour at the bottom of the picture is represented by a necrotic mass. × 130.

FIG. 19.—Walker tumour, 12 days old, 6 days after 1000 r, 20 per cent protein diet. The capsule is represented by a thick fibrous connective tissue which surrounds the necrotic tumour mass. × 130.

FIG. 20.—Walker tumour 9 days old, 3 days after 1000 r, 5 per cent protein diet. The capsule has undergone only slight fibrosis and contains many scattered tumour cells. At the bottom of the picture, the tumour parenchyma has broken down into a necrotic mass. × 130.

FIG. 21.—Walker tumour, 12 days old, 6 days after 1000 r, 5 per cent protein diet. The tumour bed is oedematous and shows little fibrosis. The tumour parenchyma is well demarcated and contains active tumour cells. × 130.

FIG. 23.—Walker tumour, ($H_1$), 20 per cent protein diet after 3000 r. The tumour boundary is sharply defined, and the connective tissue of the capsule is undergoing fibrosis. × 72.

FIG. 24.—Walker tumour ($L_1$), 5 per cent protein diet after 3000 r. The boundary of the tumour parenchyma is irregular and the connective tissue of the capsule shows only slight fibrosis. × 72.

FIG. 25.—Walker tumour ($H_2$), 20 per cent protein diet after 4000 r, showing the necrotic tumour mass on the right and the well differentiated fibrous capsule. × 72. (Compare Fig. 26).

FIG. 26.—Walker tumour ($L_2$), 5 per cent protein diet after 4000 r. There is a certain degree of pycnotic degeneration in the tumour and fibrosis in the tumour bed. × 72.

FIG. 27.—The same tumour ($L_2$), showing the active tumour cell island which lies beyond the periphery of the tumour which occupies the bottom right hand corner. × 190.

FIG. 28.—The same tumour ($L_2$) at a higher magnification to show that tumour cells which were scattered in the loose connective tissue capsule form foci of renewed activity after irradiation. × 300.

2 3 4 5

Devik, Elson, Koller and Lamerton.

8

9

Devik, Elson, Koller and Lamerton.

10

11

Devik, Elson, Koller and Lamerton.

Devik, Elson, Koller and Lamertón.

18
19
20
21

23

24

25

Devik, Elson, Koller and Lamerton.

26

27

28

Devik, Elson, Koller and Lamerton.

breakages in the chromosomes are followed by a rejoining of the broken ends in a new configuration. The presence of small, supernumerary nuclei called " micronuclei " in the resting cells is a third indication of injury. These micronuclei represent acentric chromosome fragments, which were left behind in the cytoplasm at telophase, and not included in the daughter nuclei.

A comparison has been made of the frequency of tumour cells with various cytological abnormalities, such as chromosome fragments, bridges and micronuclei at different times after irradiation of a six-day old Walker tumour in rats kept on high and low protein diets. The effects of two different doses of radiation 100 r and 300 r were studied. The data obtained (Table I) do not represent the full extent of the radiation injury resulting from any particular treatment, since an unknown proportion of injuries undergo restitution before the cytological examination is made and are thus lost for analysis. Moreover, the acentric chromosome fragments can only be seen when they lie freely in the cytoplasm, and consequently those fragments which are mixed up with the normal chromosomes and moved to the pole escape detection. Such an event is indicated by the absence of fragments in cells with chromosome bridges. Thus the number of fragments seen and recorded is often less than the number of fragments originally induced. A similar difficulty arises when we attempt to estimate the true extent of the radiation injury to cells by the number of micronuclei, since some of the chromosome fragments may be included in the daughter nuclei and some of the micronuclei can contain more than one fragment. Therefore the number of micronuclei within the cell cannot be considered as a reliable criterion of the primary radiation injury. On the assumption, however, that the chance for such events is the same in all the samples, it is justifiable to use the quantitative differences shown by the data as a basis for comparing differences in cell behaviour which may be induced by maintaining the animals on different diets.

The data of Table I shows that the greatest amount of injury was found in tumour samples taken 12 hours after irradiation, suggesting that these samples contained cells which were, at the time of irradiation in the most sensitive period of the mitotic cycle. It is inferred on experimental evidence, derived from x-rayed pollen-grains and root-tips cells (Darlington and LaCour, 1945) that the most sensitive period coincides with the duplication of the chromosome filament, which process marks the beginning of prophase. Since the injury in the cells is at a maximum at 12 hours after both 100 and 300 r, we may conclude that the duration of mitotic suppression does not differ over this range of dosage.

The number of cells with chromosome injuries and with micronuclei was found to be already quite high in the 6 hours' sample, suggesting that the time interval between treatment and the first appearance of chromosome fragments may be shorter than 6 hours. Therefore some rats were killed 4 hours after treatment, but although both chromosome fragments and bridges were observed in this early sample, their frequency could not be estimated because the so-called " physiological " effects of radiation grossly interfered with the analysis (Marquardt, 1938; Koller, 1943). These physiological effects are characteristic features of cells which were in mitosis during the radiation. Such cells show stickiness and clumping of chromosomes, which makes the identification of the true chromosome bridges and fragments very difficult. For this reason the 4 hours' sample could not be used for quantitative analysis.

The number of resting cells with micronuclei increases up to 24 hours after treatment, but at 72 hours such cells have almost disappeared from the tumour. These cells, whose nucleus is deficient in chromosome material, are now seen as degenerating cells which will eventually die.. Tumour cells which escaped radiation injury also now begin to divide and cause an increase in the relative number of normal cells.

Comparison of the results obtained at different times after irradiation with both 100 r and 300 r (Table I), in all samples except those taken after 12 hours, shows no significant difference in the number of cells injured or the number of chromosome fragments per cell, between animals maintained on high or low protein diets. The samples taken 12 hours after radiation, however, suggested the possibility of slightly different sensitivity (Table I, Column VIII). A repeat experiment was therefore carried out, but the results did not confirm this suggestion (Table II).

The inconsistent behaviour observed in the 12-hour samples may be related to the fact that all the mitotic cells in this sample represent those cells which were in their most sensitive state when irradiated. Cell behaviour in this stage is more readily affected by environmental factors than at other stages (Koller, 1946). Rigid control of environmental factors was not feasible since the experiments were of necessity carried out at different times and the rats used differed in age, weight, sex and genetic constitution. It was, therefore, felt that the results obtained with the 12-hour samples could not be relied upon and should be disregarded in view of the consistent results obtained with all the other samples. We may conclude, therefore, that no definite difference in the sensitivity of tumour cells to radiation has been shown to exist between rats maintained on high or low protein diets.

Since particular chromosome configurations enable us to distinguish between the first and second mitosis after X-radiation, the duration of the inter-mitotic (or "resting") period of the active tumour cells can be estimated. It is thus possible to compare the length of the inter-mitotic period on the two diets in order to find whether the duration of the mitotic cycle is altered by the diet or not. It was found that 48 hours after 100 or 300 r some cells in mitosis showed chromosome configurations which clearly indicated that they were now undergoing a second mitosis (Fig. 16, 17). It can be assumed that cells which show the greatest amount of injury are those which at the time of irradiation were at the beginning of prophase of mitosis. In the x-rayed Walker carcinoma the greatest amount of injury is found in the 12-hour sample and consequently the duration of mitosis from prophase to the end of telophase cannot be longer than 12 hours. The cycle of the second division (from prophase to telophase) would occupy another 12 hours, thus leaving 20 hours for the duration of the inter-mitotic period, if an allowance of 4 hours is made for the time lost by the radiation-induced mitotic suppression. Thus we may conclude that the active cells in the Walker carcinoma have a mitotic cycle of the order of 32 hours (20 hours spent in resting and 12 hours in mitosis); this agrees with data obtained in similar experiments in which irradiation is replaced by radiomimetic chemicals, such as the nitrogen mustards (Koller, unpublished communication).

From the observations of the frequency of cells in second mitosis 48 and 72 hours after irradiation with 100 or 300 r there was no indication that diet affects the duration of the mitotic cycle.

We must conclude, therefore, that the data obtained in the cytological analysis do not demonstrate an influence of diet on the sensitivity of tumour cells to radiation.

### *Histological Effects.*

*Effect of a single dose of* 1000 *r.*

In rats maintained on the high (20 per cent) protein diet it was found that 3 days after treatment with a dose of 1000 r applied direct to the tumour there was a great reduction in the number of dividing tumour cells, and at the same time a distinct process of fibrosis was in progress in the surrounding connective tissue (Fig. 18). At 6 days after the radiation treatment tissue fibrosis was much more developed, and the connective tissue capsule could be clearly distinguished from the actual tumour, since no tumour cells were present in this capsule zone. The tumour itself is enclosed within this fibrous capsule and shows widespread necrosis (Fig. 19). It does, however, contain islands of active tumour cells, which, since the growth-inhibiting effect of a single dose of 1000 r is usually only temporary, no doubt represent centres from which the renewed growth of the tumour develops.

In the animals receiving the 5 per cent protein diet, the loosely organized connective tissue capsule, 3 days after 1000 r, was not undergoing fibrosis to the same extent as the capsule in the 20 per cent protein diet animals. Very often it shows a slight oedema and contains numerous tumour cells (Fig. 20). Six days after irradiation the tumour boundary which was previously very ill-defined, becomes more distinct, presumably owing to the fact that some of those tumour cells which were scattered in the connective tissue network and have survived the radiation treatment, have now undergone proliferation and brought about the formation of a new continuous tumour parenchyma. At the periphery of the tumour numerous dividing cells are now seen (Fig. 21).

*Effect of fractionated dosage (total dose* 4000 *r).*

While a single dose of 1000 r may cause considerable inhibition of growth of the Walker rat carcinoma, and the inhibition may last for several days, complete regression of the tumour rarely occurs. The experiments with the single dose have shown, however, that at least in the 20 per cent protein diet animals, whilst the extent and rate of histological changes induced in the capsule or tumour bed is very favourable, it is insufficient to enable the animal to effect a cure. It is obvious, therefore, that the amount of radiation injury in the tumour cells must be increased without at the same time adversely affecting the favourable tissue response. It was therefore decided to increase the dose to 4000 r, which is of the order employed in the radiotherapy of human cancer, and a suitable method of fractionation for applying this dose was devised based on observations of the response of tumour capsule and tumour bed obtained in the single dose experiments. The method employed can be illustrated as follows : 1000 r - - - 500 r, 500 r - - - 500 r, 500 r - - - 500 r - - - 250 r - - - 250 r, in which - - - represents two consecutive days on which no treatment was given. By such treatment complete regressions have been obtained in nearly 90 per cent of the animals maintained on the 20 per cent, but in only about 15 per cent of those kept on the 5 per cent protein diet (Elson and Lamerton, 1949).

For the histological investigations 15 rats were treated with this fractionated dose, 7 of them being maintained on the 20 per cent and 8 on the 5 per cent protein diet. The growth of the irradiated tumours is represented in Fig. 22. Two rats of each diet group $H_1$, $H_2$ (High protein) and $L_1$, $L_2$ (low protein) were selected for histological examination ; of the remaining rats 4 out of the 5 main-

FIG. 22.—Growth of Walker tumour treated with X-radiation ; extended treatment with initial high dose (1000 r) ; total dose 4000 r. $H_1$ and $H_2$ indicate animals maintained on high protein diet and $L_1$ and $L_2$ animals maintained on low protein diet at the time they were killed for histological examination. ——— Rats fed on high (20 per cent) protein diet ; - - - - - rats fed on low (5 per cent) protein diet.

tained on the 20 per cent protein diet were cured, whilst only one cure was effected in the group of 6 animals receiving the low protein diet. From similar results obtained in previous experiments (Elson and Lamerton, 1949) and from a comparison of the growth curves of the tumours selected for histological investigation with those of the remainder of the groups shown in Fig. 22 it is reasonable to suppose that animals $H_1$ and $H_2$ on the high protein diet would have been cured, whereas $L_1$ and $L_2$ maintained on the low protein diet would not.

The tumours of the animals maintained on both diets were about the same size when the animals were killed for histological examination. Those of the high protein diet animals ($H_1$ and $H_2$), however, had grown to a considerable maximum size and were then regressing, whilst those of the low protein diet animals ($L_1$ and $L_2$) had shown earlier retardation of growth, but were beginning to assume a more rapid growth rate. The animals $H_1$ and $L_1$ had received a total dose of 3000 r (in 5 fractions), whereas $H_2$ and $L_2$ had received the full total dose of 4000 r (in 8 fractions).

In the rat $H_1$ the connective tissue capsule round the tumour was found to have undergone fibrosis, forming a very distinct boundary to the tumour. The solid continuous tumour parenchyma showed widespread cell degeneration, indicating that serious injury had been inflicted on a great number of tumour cells (Fig. 23). In the low protein diet animal $L_1$, on the other hand, numerous tumour cells were found scattered throughout the capsule zone in which fibrosis was much less marked than in $H_1$, thus making the tumour boundary very ill-defined (Fig. 24).

These differences between animals maintained on high and low protein diets became more marked after the full dose of 4000 r. In animal $H_2$ extremely intense fibrosis of the capsule was found, the damaged tumour tissue was disintegrating, and reabsorption of the necrotic tumour mass was taking place; tumour destruction and elimination is in fact in progress and should eventually lead to cure of the animal (Fig. 25).

In the low protein diet animal $L_2$ the tumour cells constituting the parenchyma had undergone " over differentiation " and pycnotic degeneration as a result of radiation injury to the chromosomes (Fig. 26). In the loosely fibrosed capsule zone, however, those tumour cells which were either undamaged or had managed to recover from the radiation injury were beginning to form islands of new tumour growth (Fig. 27, 28).

### DISCUSSION.

In assessing the response of tumours to radiation two effects must be considered : (1) The initial inhibition of tumour growth and (2) the elimination of the inhibited tumour and cure of the animal. In work with the Walker rat carcinoma 256 it has previously been shown that the diet of the animal has a definite influence on each of these processes (Elson and Lamerton, 1949). The object of the present investigation was to determine the biological basis of these diet effects.

Dealing first with process (1), it has been shown that maintenance of the animal on a low protein diet favours the immediate growth inhibitory response of the implanted Walker rat carcinoma 256 to radiation. It might be thought that this diet effect is related to an increased sensitivity of dividing tumour cells to radiation induced in some manner by the poor protein diet. The cytological investigation reported here has, however, yielded no evidence of any such increased sensitivity. Furthermore, we do not feel that such differences as have been revealed in the course of the histological investigation of the tumour bed reported here offer any very satisfactory explanation of the greater immediate tumour response in the low protein diet animals, and we are inclined to believe that some more fundamental biochemical relation is involved.

Investigations with carcinogenic chemicals have suggested that these substances may inhibit cellular growth by interfering with the normal processes of protein synthesis (Elson and Warren, 1947 ; Elson, 1949). If this is the case it might be expected that animals in which the full capacity for protein synthesis is already restricted by a deficient protein diet would probably show an immediate growth inhibitory response of both animal and tumour to treatment. Animals which in their diet were supplied with ample protein to furnish more than sufficient amino acid " building blocks " for the full efficiency of protein synthesis would not be expected to show such an immediate reaction. It is not unlikely that X-radiation may act in a similar way and in order to throw more light on this problem the protein metabolism of irradiated animals maintained under controlled nutritional conditions is now being investigated.

Considering now process (2) the histological investigations reported here suggest a feasible explanation of the dietary effects. In the radiation treatment of the Walker rat carcinoma a high protein diet has been shown to be of great assistance to the animal in the process of elimination of the inhibited tumour and the replacement of the tumour area by healthy tissue. The histological analysis has revealed that in the case of the implanted Walker rat carcinoma 256 the main effect of the high protein diet is to ensure the development of a well organized, extensive capsule of connective tissue around the growing tumour. In an untreated tumour this capsule, particularly in the earlier stages, may assist the growth of the tumour by forming a " tumour bed " well equipped to supply its nutritional requirements. Under the action of radiation, however, the natural tendency of the connective tissue capsule towards fibrosis becomes much enhanced. Thus the tumour is now enclosed within a firm fibrous capsule and, owing to the cellular damage caused by the radiation, breaks down into a necrotic mass and will eventually be eliminated and replaced by healthy tissue.

Animals maintained on a low protein diet usually fail to form such a well developed tissue capsule round the tumour implant. The tumour is thus more diffuse and although it may at first show a marked response to radiation treatment the ill-defined tissue capsule zone does not undergo fibrosis in the same way as does the firm capsule of the high protein diet animals. The tumour may stop increasing in size, but does not usually undergo marked regression. Although its main mass usually becomes necrotic, new tumour growth often develops in the incomplete capsule zone, from cells which have escaped the lethal action of the radiation.

Hence it appears that some factor or factors required for the efficient development of a connective tissue capsule are not provided in adequate amounts if the animals are maintained on a diet deficient in protein. The effect of addition to the low protein diet of substances such as amino acids, particularly cystine and methionine, amino sugars, etc., which may conceivably be involved in the process of connective tissue formation is being investigated. A knowledge of how connective tissue reactions are influenced by dietary factors clearly has an importance well beyond the field of investigation reported here.

Although our investigation has so far only been carried out on a particular animal tumour, the data obtained have a bearing on the radiotherapy of human cancer, and in particular emphasize further the importance of the tumour bed reaction, already revealed by previous work. Koller and Smithers (1946), taking into consideration the connective tissue reaction, devised treatment methods for epitheliomata, in which the total dose is of the order of 3000 r, far below the so-called " standard tumour lethal dose." Also it has been shown by Jolles and Koller (1950) that the tumour bed reaction can be influenced by a method of fractionation of the dose in space as well as time to give favourable therapeutic results.

The present animal experiments have shown that the character and organization of the tumour bed can be influenced by nutritional factors, and suggest that suitable regulation of nutritional conditions, such as, for instance, supplying a high protein diet, or supplementing the diet by protein hydrolysates, etc., and by increasing protein anabolism by hormone administration, etc., may lead to improvements in clinical response.

### SUMMARY.

The effect of the protein content of the diet on the establishment and growth of implants of the Walker rat carcinoma 256, and on the response of this tumour to X-radiation, has been investigated.

A subcutaneous graft of tumour tissue causes an inflammatory reaction to develop around the implant. This is followed by the formation of a capsule of connective tissue into which tumour cells from the implant migrate and undergo mitosis, thus establishing the growing tumour.

The nature and extent of the inflammatory reaction has been found to be similar in animals maintained on both high (20 per cent) and low (5 per cent) protein diets, but in the low protein diet animals it persists for a longer period after implantation owing to slower and less complete development of the connective tissue capsule.

In assessing the response of the tumour to radiation two effects have been considered, (*a*) the initial inhibition of tumour growth, which is favoured by a low protein diet, and (*b*) the elimination of the inhibited tumour which is favoured by a high protein diet.

Cytological investigation has revealed no evidence of increased sensitivity to radiation of tumour cells in animals maintained on a low protein diet.

Histological investigation has suggested that the favourable effect of a high protein diet on the elimination of the tumour is related to the development of the well organized capsule of connective tissue around the growing tumour. Radiation treatment, besides inhibiting division of the tumour cells, also increases the fibrosis in this capsule, which aids in the eventual elimination of the damaged tumour.

These investigations draw attention to the important role of tumour environment in the radiation treatment of cancer.

We wish to express our thanks for grants supporting this investigation from the British Empire Cancer Campaign, the Anna Fuller Fund, the Jane Coffin Childs Memorial Fund for Medical Research and the U.S. Public Health Service ; also for facilities provided by Imperial Chemical Industries Limited.

Experiment II in Table II was done by Mr. S. H. Revell (May, 1949), to whom we are greatly indebted.

### REFERENCES.

ALGIRE, G. H., AND CHALKEY, H. W.—(1945) *J. nat. Cancer Inst.*, **6**, 73.

DARLINGTON, C. D., AND LA COUR, L. F.—(1945) *J. Genet.*, **46**, 180

ELSON, L. A.—(1949) *Symp. Soc. exp. Biol.*, **3**, 327.

*Idem* AND LAMERTON, L. F.—(1949) *Brit. J. Cancer*, **3**, 414.

*Idem* AND WARREN, F. L.—(1947) *Ibid.*, **1**, 86.

*Idem*, GOULDEN, F., AND WARREN, F. L.—(1947) *Ibid.*, **1**, 80.

JOLLES, B., AND KOLLER, P. C.—(1950) *Ibid.*, **4**, 77.

KOLLER, P. C.—(1943) *Proc. Roy. Soc. Edin.*, **61**, 398.—(1946) *Brit. J. Radiol.*, **19**, 393.

*Idem.* AND SMITHERS, D. W.—(1946) *Brit. J. Radiol.*, **19**, 89.

MARQUARDT, H.—(1938) *Z. Bot.*, **32**, 401.